# The uS10c-BPG2 module mediates ribosomal RNA processing in chloroplast nucleoids

**DOI:** 10.1093/nar/gkae339

**Published:** 2024-04-30

**Authors:** Xueping Sun, Meenu Singla-Rastogi, Jingwen Wang, Chuanzhi Zhao, Xingjun Wang, Pengcheng Li

**Affiliations:** Institute of Crop Germplasm Resources (Biotechnology Research Center), Shandong Academy of Agricultural Sciences; Shandong Provincial Key Laboratory of Crop Genetic Improvement, Ecology and Physiology, Jinan, Shandong 250100, PR China; College of Life Sciences, Shandong Normal University, Jinan, Shandong 250014, PR China; Department of Biology, Indiana University, Bloomington, IN 47405, USA; Institute of Crop Germplasm Resources (Biotechnology Research Center), Shandong Academy of Agricultural Sciences; Shandong Provincial Key Laboratory of Crop Genetic Improvement, Ecology and Physiology, Jinan, Shandong 250100, PR China; College of Life Sciences, Shandong Normal University, Jinan, Shandong 250014, PR China; Institute of Crop Germplasm Resources (Biotechnology Research Center), Shandong Academy of Agricultural Sciences; Shandong Provincial Key Laboratory of Crop Genetic Improvement, Ecology and Physiology, Jinan, Shandong 250100, PR China; Institute of Crop Germplasm Resources (Biotechnology Research Center), Shandong Academy of Agricultural Sciences; Shandong Provincial Key Laboratory of Crop Genetic Improvement, Ecology and Physiology, Jinan, Shandong 250100, PR China; Institute of Crop Germplasm Resources (Biotechnology Research Center), Shandong Academy of Agricultural Sciences; Shandong Provincial Key Laboratory of Crop Genetic Improvement, Ecology and Physiology, Jinan, Shandong 250100, PR China; Department of Biology, Indiana University, Bloomington, IN 47405, USA

## Abstract

In plant chloroplasts, certain ribosomal proteins (RPs) and ribosome biogenesis factors (RBFs) are present in nucleoids, implying an association between nucleoids and ribosome biogenesis. In Arabidopsis, the YqeH-type GTPase Brassinazole-Insensitive Pale Green2 (BPG2) is a chloroplast nucleoid-associated RBF. Here, we investigated the relationship between nucleoids and BPG2-involved ribosome biogenesis steps by exploring how BPG2 targets ribosomes. Our findings demonstrate that BPG2 interacts with an essential plastid RP, uS10c, in chloroplast nucleoids in a ribosomal RNA (rRNA)-independent manner. We also discovered that *uS10c* is a haploinsufficient gene, as the heterozygous deletion of this gene leads to variegated shoots and chlorophyll aggregation. uS10c is integrated into 30S ribosomal particles when rRNA is relatively exposed and also exists in polysome fractions. In contrast, BPG2 exclusively associates with 30S ribosomal particles. Notably, the interaction between BPG2 and 30S particles is influenced by the absence of uS10c, resulting in BPG2 diffusing in chloroplasts instead of targeting nucleoids. Further, our results reveal that the loss of BPG2 function and the heterozygous deletion of *uS10c* impair the processing of 16S and 23S-4.5S rRNAs, reduce plastid protein accumulation, and trigger the plastid signaling response. Together, these findings indicate that the uS10c-BPG2 module mediates ribosome biogenesis in chloroplast nucleoids.

## Graphical Abstract

**Graphical Abstract:**
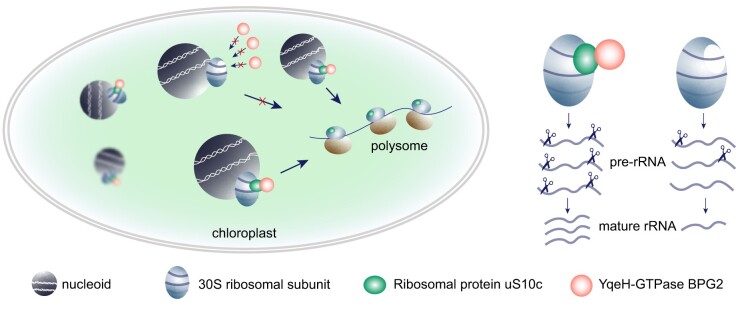

## Introduction

Ribosomes are a type of ribonucleoprotein complex responsible for protein production in both eukaryotic and prokaryotic cells. Ribosome biogenesis initiates with the transcription of ribosomal RNA (rRNA). The nascent rRNA precursor (pre-rRNA) then undergoes a series of intricately orchestrated processes, including exo- and endo-nucleolytic processing, modification, rotation, and interaction with ribosomal proteins (RPs) (1–3). Most of these steps require the involvement of non-ribosomal proteins known as ribosome biogenesis factors (RBFs) (4,5), such as ATPases (in eukaryotic cells), GTPases (in both eukaryotic and prokaryotic cells), RNA helicases, RNA modification enzymes, and scaffold proteins (in eukaryotic cells).

In eukaryotic cells, the transcription and initial processing of rRNA take place in the nucleolus, followed by subsequent maturation steps that occur successively in the nucleoplasm and cytoplasm (2,6). Prokaryotic cells house their genetic materials in highly condensed complexes called nucleoids. In *Escherichia coli*, although most mature ribosomes do not colocalize with nucleoids (7), nascent 16S rRNA precursors (pre-16S rRNAs) are found to be associated with nucleoids (8), implying that the initial steps of ribosome biogenesis occur in the vicinity of nucleoids. Semi-autonomous organelles, such as mitochondria and chloroplasts/plastids, possess prokaryotic-type ribosomes. In mammalian mitochondria, ribosome biogenesis starts co-transcriptionally near nucleoids and continues in the RNA granules (9,10). However, in plant chloroplasts, the exact location for ribosome biogenesis remains unclear.

In *E. coli*, the nucleoid undergoes rapid and dynamic organizational adjustments in a DNA-replication-independent manner (11). In plant chloroplasts, the numbers and distribution patterns of nucleoids change across different chloroplast developmental stages (12,13). In young tissues, nucleoids are typically stacked or organized in a circular pattern along the periphery. In mature leaves, nucleoid patterns vary from randomly distributed spots to a well-organized circular arrangement. Nucleoid organization is regulated by proteins associated with transcriptionally active chromosomes (TACs), which represent the core fraction of nucleoids (14,15). In addition to organizing nucleoids, TAC-associated proteins are typically involved in processes including DNA replication, transcription, and chromatin remodeling. Interestingly, a subset of RPs is identified in the TAC fraction (14). Another study focusing on the chloroplast proteome of maize leaves also revealed that RPs and RBFs are overrepresented in the nucleoid fraction (16). Consistent with these proteomic results, the distribution patterns of some chloroplast RBFs detected by microscopy resemble those of nucleoids in chloroplasts. For instance, CMAL (Chloroplast MraW-Like), an rRNA methyltransferase responsible for chloroplast pre-rRNA processing, shows a distribution pattern resembling nucleoids and colocalizes with the nucleoid TAC-associated protein pTAC5 (17). Double Era-like GTPase (DER) can directly bind to both 23S and 16S rRNAs *in vitro* and is involved in chloroplast rRNA processing. Fluorescent protein fused DER forms distinct particles in chloroplasts and colocalizes with a nucleoid TAC-associated protein referred to as S1 domain-containing Transcription-Stimulating Factor (STF) (18). RAP, the sole octotricopeptide repeat protein in Arabidopsis, binds to the 5′-region of pre-16S rRNA and participates in its processing. RAP is also found to colocalize with nucleoids (19). These observations together suggest that certain assembly steps of chloroplast ribosomes might occur around nucleoids (20).

Ribosome biogenesis is an energy-intensive process (21), requiring the involvement of many energy-consuming enzymes. Guanosine triphosphate hydrolases (GTPases) represent a major class of these enzymes during ribosome assembly (5,22,23). In bacteria, several types of GTPases are involved in ribosome assembly, including RbgA (ribosome biogenesis GTPase A), Obg, YsxC, Der, Era (*E. coli ras*), RsgA (ribosome small-subunit-dependent GTPase A), and YqeH (22). A recent study on the ribosome interactome in *Chlamydomonas* chloroplasts has revealed that these GTPase counterparts play conserved roles in ribosome biogenesis (24). In Arabidopsis, *Brassinazole*-*insensitive*-*Pale Green 2* (*BPG2*) encodes a chloroplast-localized YqeH-type GTPase. Similar to its bacterial ancestor (25,26), BPG2 is involved in the processing of 16S rRNA in chloroplasts (27). In the mitochondria of yeast and humans, Mitochondrial Ribosome-Associated GTPase 3 (MTG3) is a counterpart of BPG2. In yeast, ScMTG3 is required for the maturation of the 15S rRNA (28). In human cells, HsMTG3 specifically interacts with the small ribosomal subunit, and its absence severely affects mitochondrial protein synthesis. Notably, HsMTG3 is associated with the mitochondrial nucleoid fraction (29). In this study, we found that BPG2 also targets nucleoids in chloroplasts. Therefore, we aimed to explore the relationship between nucleoids and BPG2-mediated ribosome biogenesis steps in chloroplasts by investigating how BPG2 interacts with ribosomes.

## Materials and methods

### Plant materials and growth conditions

The Arabidopsis Col-0 ecotype was employed as the wild type (WT). The *bpg2-2* mutant has been described in a previous study (27). To create the *us10c* mutants, the *YAO* promoter-driven CRISPR-Cas9 system was used (30). To analyze the mutation sites, total DNA was extracted from T1 plants and amplified by PCR with specific primers for *uS10c*. After sequencing, the results were decoded with DSDecodeM, a web-based software (31). To generate the double mutant of *bpg2 us10c*/+, pollen from *us10c-1*/+ was smeared on the stigmas of *bpg2-2*. To generate the double mutants of *gun1-101 bpg2-2* and *gun1-101 us10c-1*/+, pollen from *gun1-101* was smeared on the stigmas of *bpg2-2* and *us10c-1*/+, respectively. The generated F1 plants were self-crossed, and subsequent F2 plants were further selected by PCR and phenotypic analysis.

To construct the estradiol-inducible *us10c* RNAi vector, a 480-bp fragment (1–480) from the coding region of *uS10c* was cloned into two opposite orientations in the pHANNIBAL vector. The entire RNAi cassette was then transferred into the estradiol-inducible vector pLB12. To obtain the *35S:uS10c-HA* vector, the coding sequence of *uS10c* combined with the HA-tag and driven by the *35S* promoter was inserted into the pCambia2300-35S vector. For the construction of the *uS10c:uS10c-GFP* vector, the coding sequence of *uS10c* (TAA free) combined with that of *GFP* and driven by the native promoter of *uS10c* (1753 bp) was cloned into the pCambia2300 vector. For generating transgenic plants, the above plasmids were transformed into *Agrobacterium tumefaciens* (GV3101), and the Agrobacterium-mediated floral-dip method was employed. For selecting transgenic plants expressing *uS10c:uS10c-GFP* in *us10c-1*/+ background, kanamycin (50 mg/l) was used for the preliminary screening of the T1 population. The kanamycin-resistant plants were further tested with PCR combined with Sanger sequencing.

For seed germination, sterilized seeds were placed on solid 1/2 Murashige and Skoog (MS) medium (pH 5.7) containing 1% (w/v) sucrose. After growing for 7–10 days, the seedlings were transferred to soil. All plants were grown in a phytotron at 22–24°C with a 16-h light/8-h dark cycle.

### Chloroplast isolation and staining with Hoechst 33342

Arabidopsis mesophyll protoplast isolation and transformation were performed according to the protocol from Jen Sheen's lab (32). For protoplast transformation, the pBSK-35S-GFP/mCherry vector was employed. For the isolation of intact chloroplasts from the transformed protoplasts, the Minute chloroplast isolation kit (Invent Biotechnologies, CP-011) was used. The isolated chloroplasts were then incubated with 1 μg/ml Hoechst 33342 (Thermo Fisher) for 30 min before confocal microscopy. Detailed methods and parameters for confocal microscopy have been described in our previous studies (33,34).

### Yeast-2-hybrid assays

A *GAL4*-based Matchmaker™ Gold Yeast Two-Hybrid System kit (630489, Clontech, Takara Bio, USA) with Mate & Plate™ Library-Universal Arabidopsis (Normalized, 630487, Clontech) was used for the BPG2 Y2H screening assays according to the provided instructions. The coding sequence of BPG2 was inserted into the pGBKT7 vector. Yeast strain Y2H Gold was employed as the bait, and strain Y187 was used as the prey. Blue colonies that grew well on quadruple dropout medium (QDO, SD/–Ade/–His/–Leu/–Trp) supplemented with X-alpha-Gal and Aureobasidin A (AbA, 200 ng/ml) were selected and sequenced using specific primers for the pGADT7 vector. To confirm the interaction between uS10c and BPG2 in yeast, the full coding sequence of uS10c was cloned from Arabidopsis cDNA and introduced into pGADT7. Plasmids of *BPG2-pGBKT7* and *uS10c-pGADT7* were co-transformed into Y2H Gold cells. The transformation mixture was then spotted onto QDO medium containing X-alpha-Gal and incubated for 3–4 days at 30°C. Primers used are listed in Table S2.

### Bimolecular fluorescence complementation (BiFC) assay

The coding sequences of *BPG2*, *uS10c* and *uS9c* were cloned from Arabidopsis cDNA and inserted into the pUC-SPYNE and pUC-SPYCE vectors, according to our experimental designs. BiFC analysis in Arabidopsis protoplasts was performed following the methods described in our previous study (34).

### *In vitro* pull-down analysis

To obtain the BPG2 protein *in vitro*, the coding sequence of *BPG2* was inserted into the pGEX-6P-1 vector. To obtain the uS10c protein, the coding sequence of uS10c was inserted into the pET28a vector. These vectors were then introduced into the *E. coli* strain Rosetta DE3 (Invitrogen). To express GST-BPG2 fusion protein, 0.5 mM isopropyl-β-d-thiogalactoside (IPTG) was added to the bacterial culture (OD_600_ = 0.5) and incubated for 10 h at 16°C. The bacterial cells were collected by centrifugation at 5000 *g* for 10 min at 4°C and resuspended in phosphate-buffered saline (PBS, pH 7.4) containing a protease inhibitor cocktail (Roche). Cell lysis was conducted by ultrasonication on ice. After centrifugation at 12 000 *g* for 15 min at 4°C, the GST-BPG2 protein in the supernatant was purified using a GST Spin Purification Kit (Cat. 16106, Thermo Scientific) according to the manufacturer's instructions. The protein solution was desalted with Zeba Spin Desalting Columns (Thermo Scientific), and proteins were redissolved in PBS. To express the His-uS10c recombinant protein, 0.1 mM IPTG was applied to the bacterial culture (OD_600_ = 0.5) and incubated for 12 h at 20°C. The His-uS10c protein was purified using a His-tagged protein purification kit (CW0894S, CWBIO). For the pull-down assay, purified proteins in 1X PBS (200 μl each) were mixed and incubated at 23°C for 60 min. The mixture was further incubated with Ni-NTA Magnetic Beads (v/v, 10:1, Cat. S1423S, NEB) for another 40 min at 4°C. After incubation, the beads were washed ten times with the following wash buffer (25 mm Tris–HCl, pH 7.5, 2 mm EDTA, and 100 mm NaCl). The proteins on the beads were eluted with 2× SDS-PAGE loading buffer and analyzed by immunoblotting.

### Immunoprecipitation

Total native proteins were extracted from the shoots of seven-day-old seedlings of *uS10c:uS10c-GFP*, *RAB8D:RAB8D-GFP* (34), and cpGFP transgenic plants using the lysis buffer (10 mM Tris/Cl pH 7.5, 150 mM NaCl, 0.5 mM EDTA, 0.5% Nonidet™ P40 Substitute, 0.09% sodium azide). The protein extracts were incubated with GFP-Trap Agarose (Chromotek) for 60 min at 4°C. After centrifugation (2500 *g* for 5 min at 4°C), the agarose resin beads were resuspended in wash buffer (10 mM Tris–HCl pH 7.5, 150 mM NaCl, 0.05% Nonidet™ P40 Substitute, 0.5 mM EDTA, 0.018% sodium azide). Following five repetitive wash steps, the beads were resuspended in 80 μl of two-fold SDS loading buffer and boiled for 5–10 min at approximately 95°C. The samples were then analyzed by immunoblotting. For RNase treatment, the extracts were successively incubated with RNase A (Sigma, 1 mg/ml) for 20 min and with GFP-Trap Agarose for 60 min at 4°C, followed by the procedure described above. RNA purification from the agarose resin beads was performed using TRIzol. For the RNA gel blot analysis, equal volumes of immunoprecipitated RNA were loaded (refer to the RNA gel blot method described below).

### Embryo observation

For observing the embryo phenotypes, siliques were collected and fixed with ethanol/acetic acid (9:1, v/v) for at least 48 h. After fixation, the siliques were successively incubated with 90% and then 70% ethanol, for 1 h each. They were further cleared with chloral hydrate/glycerol/water (8:1:2, w/v/v) for 2 h or until the siliques sank to the bottom of the tubes. The cleared seeds were then transferred onto a glass slide and examined with confocal microscopy using the Differential Interference Contrast (DIC) mode.

### Sucrose density gradient ultracentrifugation

For the sucrose density gradient, total proteins were extracted from three-day-old seedlings using the native extraction buffer supplied by the Minute Total Protein Extraction Kit for Plant Tissues (Invent Biotechnologies, SN-009). Approximately 1 mg of the extracted proteins was loaded onto a 4.5-ml sucrose cushion (10–55%) and then centrifuged using an SW55Ti rotor (Beckman) at 45 000 rpm for 2 h at 4°C. For the RNase treatment, the native protein extracts were incubated with 1 mg/ml RNase A for 20 min before centrifugation. For EDTA treatment, 20 mM EDTA was added to the protein samples before centrifugation. For immunoblotting, the sucrose solution after centrifugation was equally divided into 12 fractions (columns 1–12, from light to heavy). Each fraction was further separated by SDS-PAGE electrophoresis and then transferred onto polyvinylidene difluoride membranes. Proteins were detected using specific antibodies. The BPG2 antibody was produced in rabbits using amino acids 313–445 as the antigen. Antibodies for bS1c (AS15 2875), uL2c (AS15 2876), uL4c (AS15 3076) and ACTIN (AS13 2640) were all purchased from Agrisera (Sweden). The GFP and HA antibodies were obtained from Abmart (China). For analyzing the RNA profile after ultracentrifugation, total ribonucleoprotein particles and polysomes were extracted with polysome extraction buffer (0.2 M Tris–HCl pH 9.0, 0.2 M KCl, 35 mM MgCl_2_, 25 mM EGTA, 0.2 M sucrose, 1% TritonX-100 and 2% polyoxyethylene-10-tridecyl ether). After ultracentrifugation at 45 000 rpm for 2 h at 4°C, RNA was extracted with phenol/chloroform/isoamyl alcohol (25:25:1, v/v/v) and ethanol from sucrose density gradient fractions 1–12. For the RNA gel blot, each well was loaded with an equal volume (3 μl in this study) of RNA.

### RNA gel blot

Total RNA was extracted from 3 DAG (days after germination) seedlings of WT, *bpg2-2*, *us10c-1*/+ and the *bpg2 us10c*/+ double mutant using a Spectrum™ plant total RNA kit (Cat. STRN, Sigma-Aldrich). Probes were prepared using a DIG Oligonucleotide Tailing Kit (Cat. 3353583910, Roche) according to the manufacturer's instructions. For RNA-gel blot analysis, 2 μg of total RNA was separated on a 1% agarose gel prepared with 1X MOPS and 3.7% formaldehyde. After electrophoresis, the gel was stained with 1× SYBR Gold in 0.5× TBE buffer for 30 min, following which the gel was washed with 0.5X TBE before imaging for loading control. After imaging, RNA was transferred onto a Nylon membrane using the semi-dry system. The membrane with RNA was cross-linked twice at 120 000 μJ/cm^2^ for 30 s by a UVC-508 Ultraviolet Cross-linker (Ultra-Lum). After cross-linking, the membrane was prehybridized with DIG (digoxigenin) Easy Hyb solution (Cat. 11603558001, Roche) containing 0.1 mg/ml of Poly(A) at 42ºC for 40 min. It was then hybridized with DIG-labeled DNA probes (2.5 pmol/ml in DIG Easy Hyb solution containing Poly(A), Table S2) at 42ºC overnight. The membrane was successively washed with 2× Saline Sodium Citrate (SSC) buffer containing 0.1% SDS (sodium dodecyl sulfate) at room temperature (RT) twice for 5 min, then 1× SSC containing 0.1% SDS at 42ºC twice for 15 min. After washing, the membrane was incubated in blocking solution (provided by DIG Wash and Block Buffer Set, Cat. 11585762001, Roche) followed by anti-DIG antibody solution for 30 min each. The membrane was washed twice with washing buffer (0.1 M maleic acid, 0.15 M NaCl, pH 7.5, 0.3% (v/v) Tween 20) for 15 min at RT and then incubated with detection buffer (provided by DIG Wash and Block Buffer Set, Roche) for 5 min. The membrane was immersed into 200–1000 μl CDP-*Star* ready-to-use solution (Cat. 12041677001, Roche) and incubated at 37ºC for 5–10 min at dark, and the chemiluminescence signal was detected using the Bio-Rad ChemiDoc imaging system. To re-probe the membrane with other probes, it was first stripped using the stripping buffer (50% Formamide, 50 mM Tris-HCl pH 7.5, 5% SDS) twice at 80ºC for 50 min each and then washed thrice with 4× SSC buffer at RT.

### Circular reverse transcription PCR (cRT-PCR)

For RNA cyclization, approximately 10 μg of RNA was incubated at 70°C for 5 min and then immediately chilled on ice for 2 min. T4 RNA ligase (30 U), 1 mM ATP, and RNase inhibitor (40 U) were mixed with the RNA in a total volume of 50 μl. This mixture was then incubated at 37°C for 1.5 h. After the ligation, the circular RNA was precipitated with ethanol and resuspended in RNase-free water. To generate cDNA from the circular RNA, the TransScript II First-Strand cDNA Synthesis SuperMix kit (AH301, TransGen Biotech) was used along with corresponding transcription primers (Table S2). Following PCR amplification (30 cycles) with specific primers (Table S2), the DNA products were analyzed by gel electrophoresis. Subsequently, the DNA was purified and inserted into the pEasy-T vector for further sequencing.

### Data-independent acquisition mass spectrometry (DIA-MS)

Total protein was extracted from 3 DAG (days after germination) seedlings of WT, *bpg2-2*, *us10c-1*/+ and *bpg2 us10c*/+. Extracted protein (approximately 100 μg for each sample) was diluted with 50 mM NH_4_HCO_3_ at a 1:4 (v/v) ratio and digested with 2.5 μg of Trypsin at a 1:40 m/m ratio for 4 h at 37°C. The enzymatic peptides were then desalted using a Strata X column and vacuum dried. After high-pH reversed-phase separation using a Shimadzu LC-20AB HPLC system coupled with a Gemini high pH C18 column (5 μm, 4.6 × 250 mm), the dried peptides were reconstituted with mobile phase A (2% ACN, 1% FA). Following centrifugation at 20 000 *g* for 10 min, the supernatant was further separated by Thermo UltiMate 3000 UHPLC. For DDA (data-dependent acquisition) and DIA (data-independent acquisition) analyses, the peptides separated by the liquid phase were ionized using nanoESI and injected into a tandem mass spectrometer, Fusion Lumos (Thermo Fisher Scientific), in DDA and DIA detection modes, respectively. DDA data were processed using the Andromeda search engine within MaxQuant, and the identification results were used for spectral library construction. The parameters were as follows: enzyme, trypsin; minimal peptide length, 7; PSM-level FDR, 0.01; and protein FDR, 0.01. The DIA data were analyzed using iRT peptides for retention time calibration. Based on the target-decoy model applicable to SWATH-MS, false positive control was performed with an FDR of <0.01. Differential protein screening was conducted based on a fold change of >1 and a *P*-value of <0.05. Both DDA and DIA raw data are available via ProteomeXchange under the identifier PXD027861.

## Results

### BPG2 interacts with ribosomal protein uS10c in chloroplast nucleoids

**Figure 1.**
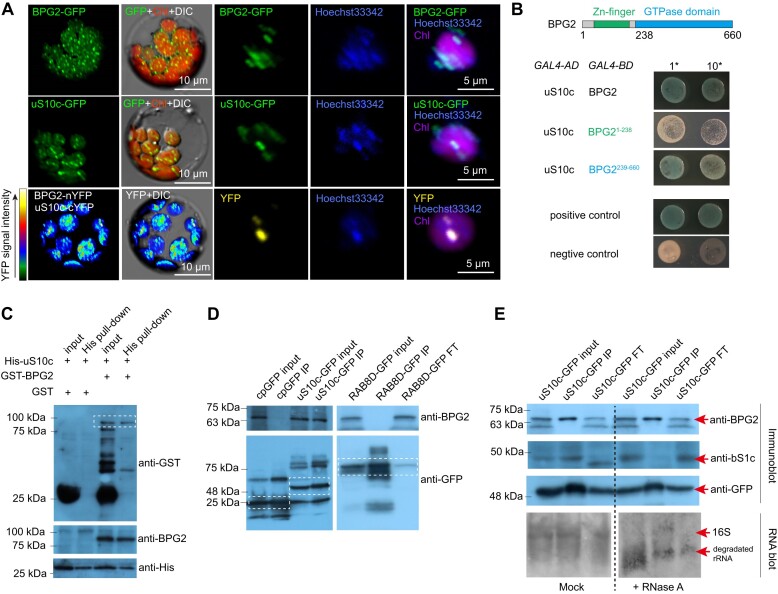
BPG2 interacts with uS10c in chloroplast nucleoids. (**A**) Subcellular localization analysis of BPG2 and uS10c, and BiFC analysis of the interaction between uS10c and BPG2. The heatmap indicates the YFP fluorescence signal intensity. The DNA-specific dye, Hoechst 33342, was used to stain nucleoids in chloroplasts. Chl, chlorophyll autofluorescence. DIC, differential interference contrast. (**B**) Yeast two-hybrid (Y2H) analysis examining the interaction of uS10c with BPG2, BPG2^1-238^, and BPG2^239-660^. Positive control, p53/SV40; negative control, Lamin/SV40. Yeast growth medium, SD/-Ade/-His/-Leu/-Trp/X-α-Gal. *GAL4-AD*, pGADT7 vector; *GAL4-BD*, pGBKT7 vector. The upper panel illustrates the protein domains of BPG2. (**C**) Pull-down analysis to study the interaction between uS10c and BPG2. Specific antibodies targeting the GST tag and BPG2 were used to detect the GST-BPG2 protein. A His_6_-specific antibody was used to detect the His-uS10c fusion protein. The white dotted box highlights the band corresponding to BPG2. (**D**) Immunoprecipitation was performed on native protein extracts from transgenic plants containing *35S:cpGFP*, *uS10c:uS10c-GFP*, and *RAB8D:RAB8D-GFP*, using GFP-trap beads. A BPG2-specific antibody was employed to detect the BPG2 protein. White dotted boxes indicate corresponding bands for cpGFP, uS10c-GFP, and RAB8D-GFP. FT, flowthrough fractions. (**E**) Immunoprecipitation from native protein extracts of *uS10c:uS10c-GFP* transgenic plants was conducted using GFP-trap beads. Before incubation with the beads, plant extracts were treated with or without RNase A. BPG2 and bS1c proteins were detected using specific antibodies. Protein bands are indicated with red arrowheads. RNA gel blot was performed with 16S rRNA-specific probe p1 (see Figure 5A for detailed information).

YqeH is a conserved GTPase that acts in ribosome biogenesis in bacteria (25), mitochondria (29) and chloroplasts (24). However, the mechanism by which YqeH interacts with ribosomes is unclear. In Arabidopsis, BPG2 is a YqeH counterpart that localizes in chloroplasts (27,35,36). In this study, we found that BPG2, labeled with Green Fluorescent Protein (GFP, BPG2-GFP), forms small punctate structures within chloroplasts (Figure 1A). This distribution pattern resembles that of chloroplast nucleoids, complexes that contain DNA (12). A colocalization analysis between BPG2-GFP and nucleoids, stained with the DNA-specific dye Hoechst 33342, confirmed that BPG2 is associated with these nucleoid structures in chloroplasts (Figure 1A).

The BPG2 protein is composed of a potential zinc-finger domain at the N-terminus and a circularly permuted GTPase domain at the C-terminus (Figure 1B). Subcellular localization studies indicated that a truncated form of BPG2 (BPG2^1-238^-GFP), which lacks the GTPase domain, displayed a diffuse distribution pattern throughout the chloroplast rather than forming distinct foci (Supplementary Figure S1). This suggests that the GTPase domain may be critical for the association of BPG2 with nucleoids. In support of this, a construct containing the GTPase domain of BPG2 (BPG2^239-660^), fused with the plastid transit peptide from RbcS1A (Rubisco Small Chain 1A, a protein targeting chloroplasts but not associated with nucleoids), was observed to target nucleoids in chloroplasts (as shown in cpBPG2^239-660^-GFP, Supplementary Figure S1).

To explore how BPG2 targets ribosomes, we employed a partner screening approach. Using the yeast two-hybrid (Y2H) assay, we identified eleven potential interacting partners, six of which were predicted to be localized in chloroplasts (Supplementary Table S1). Among these six candidates, plastid ribosomal protein S10 (referred to as uS10c) was highlighted as it is embedded in 16S rRNA at the ‘head’ of the 30S ribosomal subunit (37–39). *In vitro* pull-down experiments showed that the recombinant GST-BPG2 protein could interact with the His-tagged uS10c fusion protein (Figure 1C). Y2H results further demonstrated that BPG2^1-238^ had a limited interaction with uS10c, whereas BPG2^239-660^ exhibited a strong interaction with uS10c (Figure 1B), indicating the critical role of the GTPase domain in the interaction of BPG2 with uS10c.

Subcellular localization analysis revealed that uS10c also targets nucleoids (Figure 1A). To test whether BPG2 is associated with uS10c *in vivo*, a bimolecular fluorescence complementation (BiFC) assay was performed. The reconstituted Yellow Fluorescent Protein (YFP) signal was observed in chloroplasts containing BPG2 fused with the N-terminal fragment of YFP (BPG2-nYFP) and uS10c fused with the C-terminal segment of YFP (uS10c-cYFP, Figure 1A). In particular, the strongest signal was observed in nucleoids (Figure 1A), indicating proximity between BPG2 and uS10c *in vivo*. According to the chloroplast ribosome structure, uS10c is located adjacent to the plastid ribosomal protein S9 (referred to as uS9c) at the ‘head’ of the 30S subunit (37–39). Indeed, through conducting the BiFC assay, the reconstituted YFP signal could be detected in chloroplasts containing both uS10c-nYFP and uS9c-cYFP (Supplementary Figure S2). However, no noticeable YFP signal was observed when BPG2-nYFP and uS9c-cYFP were co-expressed (Supplementary Figure S2), suggesting that BPG2 might specifically interact with uS10c through its GTPase domain at the ‘head’ of the 30S subunit.

We further tested the interaction between BPG2 and uS10c *in vivo* using immunoprecipitation. The interacting complexes of uS10c were immunoprecipitated from native protein extracts of *uS10c:uS10c-GFP* transgenic plants using GFP-trap agarose resin beads. Here, GFP fused with a chloroplast transit peptide from RbcS1A (cpGFP) was employed as a control (40). Since uS10c is associated with nucleoids, we also employed GFP fused with the plastid translation elongation factor EF-Tu, a nucleoid-associated protein encoded by *RAB GTPASE HOMOLOG 8D* (RAB8D-GFP) as another control (14–16,34,41,42). Immunoblotting revealed the presence of BPG2 in the immunoprecipitated products of uS10c-GFP, but not in those of cpGFP or RAB8D-GFP (Figure 1D).

Based on previous findings that BPG2 associates with 16S rRNA *in vivo* (27,36), we conducted additional experiments to determine if the BPG2-uS10c interaction was dependent on 16S rRNA. We subjected plant extracts to RNase treatment before immunoprecipitation. The results showed a significant reduction in the levels of 16S rRNA and plastid ribosomal protein S1 (referred to as bS1c) in the RNase-treated uS10c-GFP immunoprecipitated products (Figure 1E). In contrast, BPG2 was still efficiently pulled down by uS10c-GFP (Figure 1E). This outcome suggests that the interaction between BPG2 and uS10c occurs independently of 16S rRNA.

### Genetic interplay analysis between *BPG2* and *uS10c*

**Figure 2.**
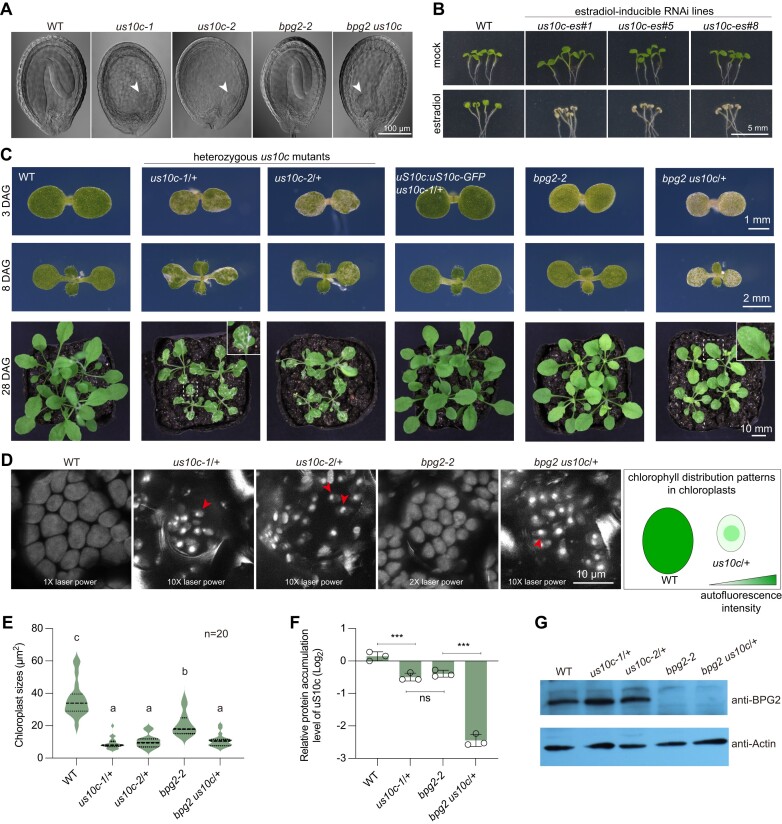
*uS10c* is a haploinsufficient gene. (**A**) Embryo phenotypes of WT, *bpg2-2*, *us10c-1*, *us10c-2* and *bpg2 us10c* at 12 days after pollination, showing that the loss of *uS10c* function results in arrested embryo development at the globular stage. (**B**) Seedling phenotypes of estradiol-inducible *uS10c* RNAi lines grown under control or 1 μM estradiol treatment conditions at 5 days after germination (DAG), with three representative lines displayed. (**C**) Shoot phenotypes of WT, *us10c-1*/+, *us10c-2*/+, *uS10c:uS10c-GFP us10c-1*/+, *bpg2-2*, and *bpg2 us10c*/+ at 3 DAG, 8 DAG and 28 DAG. (**D**) Chlorophyll distribution patterns in cotyledon chloroplasts of WT, *us10c-1*/+, *us10c-2*/+, *bpg2-2* and *bpg2 us10c*/+ at 4 DAG. The diagram highlights the differences in chlorophyll distribution between WT and *us10c*/+ mutants. The green color difference indicates the intensity of chlorophyll autofluorescence. Red arrowheads indicate chloroplasts with aggregated chlorophyll. (**E**) A violin plot shows the comparison of cotyledon chloroplast size (*n* = 20) among WT, *us10c-1*/+, *us10c-2*/+, *bpg2-2* and *bpg2 us10c*/+ at 5 DAG. One-way ANOVA was used for statistical analysis, with different letters indicating significant differences at *P* < 0.0001. (**F**) Comparison of relative protein accumulation level of uS10c among WT (with one of the log2 values set to 0), *us10c-1*/+, *bpg2-2* and *bpg2 us10c*/+. Data were obtained with DIA-MS (see Supplementary Dataset S1). Error bars represent SD (*n* = 3). Significance was assessed using the Student's *t*-test, with *** indicating *P* < 0.0001. (**G**) Immunoblotting analysis to determine the accumulation levels of BPG2 protein in 3-day-old seedlings of WT, *us10c-1*/+, *us10c-2*/+, *bpg2-2* and *bpg2 us10c*/+. Actin was employed as an internal control.

Prior studies have revealed that the knockout mutant of *BPG2*, *bpg2-2*, exhibits a pale-green shoot phenotype (27,35,36), but the physiological role of *uS10c* is unknown. We employed the CRISPR-Cas9 system to induce mutations in *uS10c*, positioning the Cas9 target site next to the chloroplast transit peptide (cTP) coding region (Supplementary Figure S3A). Two mutant alleles (referred to as *us10c-1* and *us10c-2*) were selected for further study. The *us10c-1* allele has a thymine insertion at the Cas9 target site, causing a frameshift mutation, while *us10c-2* contains base changes resulting in an amino acid switch from glutamic acid to glycine. Both mutations prevent the generation of homozygous offspring, with frequent occurrences of albino seeds in mature green siliques (Supplementary Figure S3B). These albino seeds generally contained embryos arrested at the globular stage, underscoring the critical role of uS10c in plant viability (Figure 2A). To bypass embryonic lethality, estradiol-inducible *uS10c* RNA-interference (RNAi) transgenic plants (*us10c-es*) were created. Depletion of *uS10c* expression upon estradiol treatment resulted in pronounced albinism in shoots (Figure 2B), highlighting the essential role of *uS10c* in chloroplast development.

Notably, while the heterozygous mutants, *us10c-1*/+ and *us10c-2*/+, remained viable, they exhibited noticeable variegation and deformities in their cotyledons and leaves (Figure 2C, Supplementary Figure S3C). In comparison to the wild type (WT) and the *bpg2-2* mutant, chloroplasts from these *us10c*/+ mutants were smaller in size (Figure 2D, E). Furthermore, a striking difference was observed in the distribution of chlorophyll within the chloroplasts: unlike the observation that chlorophyll is evenly distributed in WT and *bpg2-2* chloroplasts, in the majority of chloroplasts from the *us10c*/+ mutants, chlorophyll was concentrated into a distinct spot (Figure 2D, Supplementary Figure S3D), particularly in lighter-colored areas.

We did not obtain a satisfactory antibody for detecting the uS10c protein. To assess the impact of the heterozygous deletion of *uS10c* on its protein accumulation level, we conducted data-independent acquisition mass spectrometry (DIA-MS, Supplementary Dataset S1). The results showed that *us10c-1*/+ retained approximately 64.1% of the WT uS10c protein level (Figure 2F, also highlighted in Supplementary Dataset S1). Introducing a *uS10c:uS10c-GFP* fusion gene into *us10c-1*/+ rescued the abnormal phenotypes (Figure 2C), suggesting that the compromised fitness might stem from heterozygous deletion of the *uS10c* gene, a phenomenon known as haploinsufficiency (43). Interestingly, in the *bpg2-2* context, the protein accumulation of uS10c was also reduced to a level comparable to that detected in *us10c-1*/+ (Figure 2F), which might be associated with the plastid signaling (see the following results and discussion). The appearance discrepancy between *bpg2-2* and *us10c*/+ mutants suggests that the phenotype elicited by uS10c deficiency manifests in a context-dependent manner.

To test this hypothesis, we analyzed the impact of the heterozygous deletion of *uS10c* combined with loss of *BPG2* function on the plant shoot phenotype. *bpg2-2* was crossed with *us10c-1*/+ to yield offspring with both the homozygous *bpg2-2* and heterozygous *us10c-1*/+ mutations (referred to as *bpg2 us10c*/+). The absence of BPG2 protein was confirmed in the double mutant with immunoblotting (Figure 2G). According to the DIA-MS results, the double mutant retained 16.6% of the WT uS10c protein level (Figure 2D). As expected, no homozygous *us10c-1* progeny was isolated, and arrested globular embryos were observed in *bpg2 us10c*/+ progenies (Figure 2A, Supplementary Figure S3B). However, unlike *us10c-1*/+, the cotyledons of the *bpg2 us10c*/+ double mutant maintained a normal shape, though they appeared paler than those of the single mutants (Figure 2C). The rosette leaves of *bpg2 us10c*/+ displayed like the *bpg2-2* single mutant, albeit with minor variegation and a reduction in shoot size (Figure 2C, Supplementary Figure S3C). Moreover, chlorophyll became evenly distributed in most chloroplasts of *bpg2 us10c*/+ leaves (Supplementary Figure S3D). These observations indicate that the impact resulting from *uS10c* haploinsufficiency is mitigated in the *bpg2-2* context, which supports the above hypothesis.

### uS10c mediates the interaction between BPG2 and pre-30S ribosomal particles

**Figure 3.**
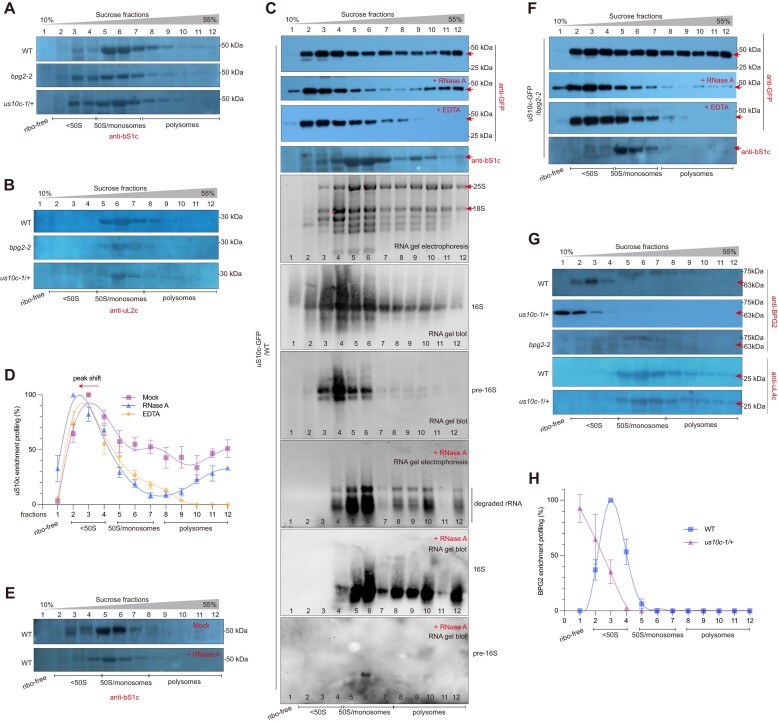
Analysis of the association of uS10c and BPG2 with chloroplast ribosomes using sucrose density gradient centrifugation. (A and B) Analysis of the migration patterns of bS1c (**A**) and uL2c (**B**) in sucrose density gradient fractions (10–55%). Total native proteins were extracted from WT, *bpg2-2* and *us10c-1*/+. (**C**) Co-migration analysis of uS10c-GFP and 16S rRNA in sucrose density gradient fractions (10–55%). Total proteins and RNA were extracted from *uS10c:uS10c-GFP* transgenic plants in the WT background. Before ultracentrifugation, plant extracts were treated with or without RNase A or EDTA. Immunoblotting was performed using anti-GFP and anti-bS1c antibodies to detect the uS10c-GFP and bS1c proteins, respectively. RNA gel blot analysis was conducted with probes specific to 16S (the p1 probe) and pre-16S rRNAs (the p2 probe; see Figure 5A for detailed information). Red arrowheads indicate the uS10c-GFP protein bands in the immunoblot results. For RNA gel electrophoresis results, 25S, 18S, and degraded rRNAs (after RNase A treatment) are marked. The intact RNA gels, stained with SYBR Gold, are presented in Supplementary Figure S4A. (**D**) Analysis of the enrichment profiling of uS10c in sucrose density gradient fractions after treatment with or without RNase A or EDTA. The uS10c protein levels were determined by the gray values of bands calculated using ImageJ software, with the highest value in each sample set to 1 (100%). (**E**) Analysis of the migration patterns of bS1c in sucrose density gradient fractions after treatment with or without RNase A. Proteins were extracted from the WT background. (**F**) Migration pattern analysis of the uS10c-GFP fusion protein extracted from the *uS10c:uS10c-GFP* transgenic plants in the *bpg2-2* background in sucrose density gradient fractions (10–55%). Native protein extracts were treated with or without RNase A or EDTA before ultracentrifugation. (**G**) Analysis of the migration patterns of BPG2 in sucrose density gradient fractions. Native proteins were extracted from WT, *us10c-1*/+ and *bpg2-2* (as a negative control). Red arrowheads indicate the BPG2 protein bands. RP uL4c migration patterns were used as controls. (**H**) Comparison of the BPG2 enrichment profiling in sucrose density gradient fractions between the WT and *us10c-1*/+ backgrounds. The BPG2 protein level in each fraction was determined by the gray value of the band calculated using ImageJ software, with the highest value in each sample set to 1 (100%).

Next, to elucidate the molecular relationship between BPG2 and uS10c during ribosome biogenesis, we investigated the association of uS10c and BPG2 with chloroplast ribosomes through sucrose density gradient centrifugation. Plastid RPs bS1c and L2 (hereafter referred to as uL2c) were utilized to indicate the enrichment patterns of the 30S and 50S subunits in sucrose density gradient fractions, respectively (39). The results showed that bS1c was present in fractions 3 to 12, with significant enrichment observed in fractions 5 and 6 (Figure 3A). In contrast, uL2c was detected in fractions 5 to 11, predominantly enriched in fraction 6 (Figure 3B). Accordingly, ribosomal particles lighter than 50S might be enriched in fractions 1 to 4. Since no rRNA was detected in fraction 1 (Supplementary Figure S4A), we designated fraction 1 as the ribosome-free fraction.

To examine the enrichment pattern of uS10c in sucrose fractions, we employed transgenic plants expressing *uS10c::uS10c-GFP* to detect the association between the uS10c-GFP fusion protein and ribosomes. The results showed that uS10c-GFP was present in fractions 2–12, with relatively higher enrichment levels in fractions 3 and 4 (Figure 3C). Against this background, the enrichment pattern of bS1c was similar to that observed in WT, with significant enrichment in fractions 5 and 6 (Figure 3A, C). The discrepancy between the patterns of uS10c and bS1c indicates that uS10c is present in lower-molecular-weight particles that lack bS1c. Additionally, the results of the RNA gel blot analysis indicated that mature 16S rRNA migrated in fractions 2 to 12, with significant enrichment levels in fractions 4–6, whereas pre-16S rRNA was detected in fractions 3–6, with a significant peak in fraction 4 (Figure 3C). The co-enrichment of uS10c and pre-16S in fractions 3 and 4 suggests that uS10c is present in pre-ribosomal particles with unprocessed 16S rRNA. These results together demonstrate the involvement of uS10c in an earlier assembly step of the 30S subunit compared to bS1c.

We further tested the association between uS10c and ribosomes after treatment with RNase A and EDTA. RNase A targets and cleaves exposed RNA, while EDTA disassembles ribosomes into subparticles. The results showed that the enrichment levels of uS10c in fractions 8–12 (hereafter referred to as polysome fractions) were reduced after RNase or EDTA treatment (Figure 3C), indicating polysome disassociation. Importantly, RNase A treatment increased the level of uS10c in the ribosome-free fraction, with the enrichment peak shifting from fraction 3 to fraction 2 (Figure 3C, D). However, EDTA treatment did not result in uS10c release into the ribosome-free fraction, although it did somewhat increase the level of uS10c in fraction 2 (Figure 3C, D). These results suggest that the uS10c-embedded pre-ribosomal particles are sensitive to RNase. Indeed, both 16S and pre-16S rRNAs were scarcely detected in fractions 2–4 following RNase treatment (Figure 3C, Supplementary Figure S4A). In contrast, the enrichment pattern of bS1c remained unchanged after RNase treatment (Figure 3E), indicating that bS1c-embedded ribosomes are more resistant to RNase. To ensure that the GFP tag did not influence the interaction between uS10c and ribosomes, we conducted a parallel experiment with uS10c fused to an HA tag (uS10c-HA). The results showed that uS10c-HA exhibited a similar pattern to uS10c-GFP in the sucrose fractions, regardless of RNase or EDTA treatment (Supplementary Figure S4B). Collectively, these findings support the hypothesis that uS10c is integrated into pre-30S ribosomes when rRNA is loosely packed, making them more vulnerable to nucleolytic attacks.

In yeast, some RBFs assist RPs in their incorporation into pre-ribosomal particles (44). Therefore, we explored whether BPG2 is involved in facilitating the association between uS10c and ribosomes. The results showed that the enrichment pattern of uS10c in sucrose fractions was not affected in the *bpg2-2* context, even after treatment with RNase A or EDTA (Figure 3F), suggesting that the association between uS10c and ribosomes is independent of BPG2.

Compared with uS10c, BPG2 was only detected in fractions 2–4, with an enrichment peak in fraction 3 (Figure 3G, H), demonstrating that BPG2 is exclusively associated with the 30S subunit. However, in the *us10c-1*/+ mutant, BPG2 was detected in fractions 1–3 and was predominantly enriched in the ribosome-free fraction (Figure 3G, H), indicating that a fraction of BPG2 could not target ribosomes. In contrast, the enrichment patterns of ribosomal proteins such as bS1c (Figure 3A), uL2c (Figure 3B), and uL4c (Figure 3G) were not affected by the heterozygous deletion of *uS10c*. These results suggest that uS10c mediates the association between BPG2 and the 30S ribosomal particles.

### uS10c is required for BPG2 targeting nucleoids

**Figure 4.**
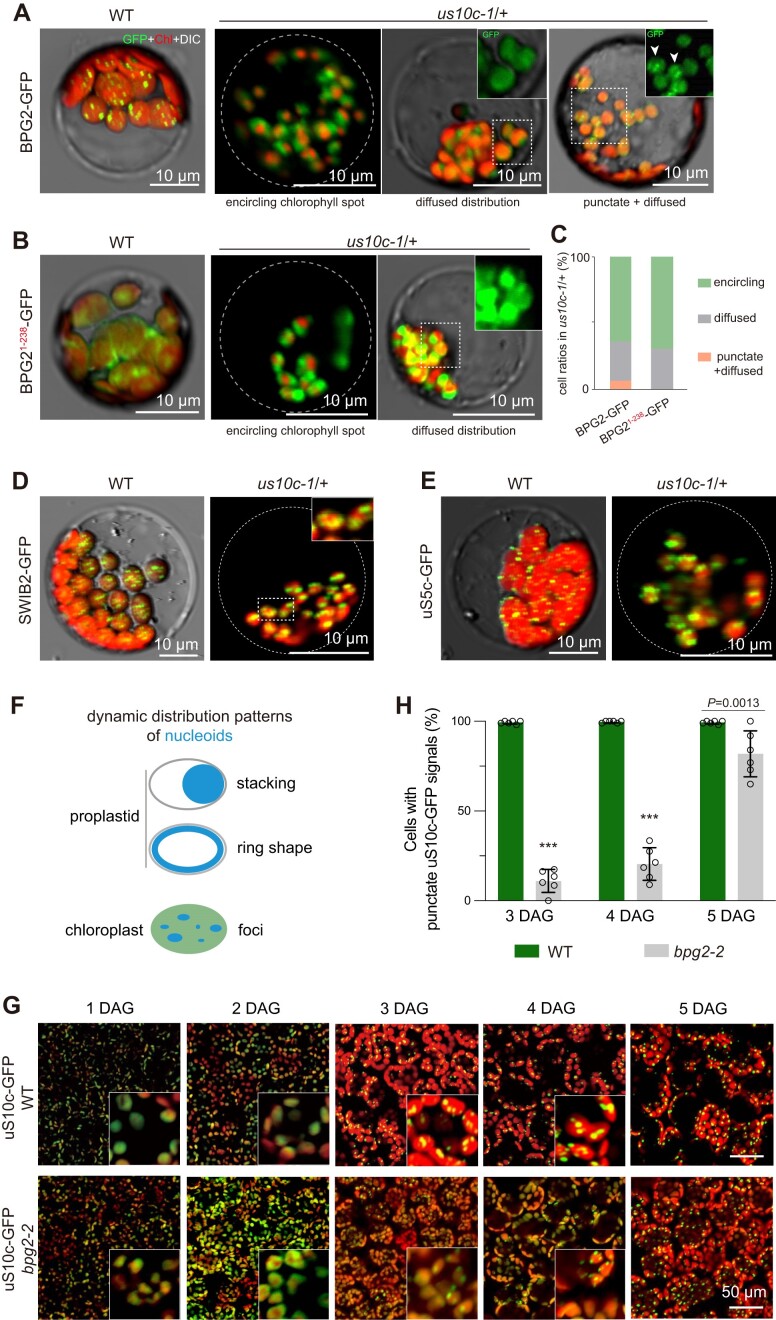
Localization analyses of BPG2 and uS10c within chloroplasts. (**A**) Analysis of the distribution patterns of BPG2-GFP within chloroplasts in cells from either the WT background or carrying the *us10c-1*/+ mutation. In the *us10c-1*/+ context, three different kinds of cells were observed regarding the distribution pattern of BPG2-GFP: 1) cells displaying BPG2-GFP fluorescence surrounding the aggregated chlorophyll; 2) cells exhibiting uniformly dispersed BPG2-GFP throughout the chloroplast; and 3) cells containing both types of chloroplasts, one with punctate BPG2-GFP fluorescence and the other with diffusely scattered BPG2-GFP. White arrowheads indicate chloroplasts showing punctate BPG2-GFP signals. (**B**) Analysis of the distribution patterns of BPG2^1-238^-GFP within chloroplasts in the WT or *us10c-1*/+ background. (**C**) Percentage of *us10c-1*/+ cells exhibiting distinct distribution patterns for BPG2-GFP and BPG2^1-238^-GFP. (D and E) Distribution pattern analysis of SWIB2-GFP (**D**) and uS5c-GFP (**E**) within chloroplasts of WT and *us10c-1*/+. (**F**) A diagram illustrating the organization of nucleoids in proplastids (or young chloroplasts) and mature chloroplasts. In proplastids, nucleoids typically appear stacked or in a ring formation, whereas mature chloroplasts feature nucleoids with a punctate distribution. (**G**) Dynamic distribution patterns of uS10c-GFP in chloroplasts of WT and *bpg2-2* during the initial days following germination (1–5 DAG). Zoom-in images are shown in corresponding panels for better observation. (**H**) Percentage of cells exhibiting the punctate uS10c-GFP organization pattern in cotyledons of WT and *bpg2-2* at 3–5 DAG. Statistical significance was determined using the Student's *t*-test (***P < 0.0001). For (A), (B), (D) and (E), single-channel images are provided in Supplementary Figure S5.

The above observations prompted us to investigate the impact of uS10c deficiency on the distribution pattern of BPG2 within chloroplasts. As mentioned above, BPG2 is associated with nucleoids in chloroplasts. However, instead of forming distinct foci as observed in WT leaf cell chloroplasts, BPG2-GFP fluorescence in most *us10c-1*/+ leaf cells was found to either encircle the aggregated chlorophyll or disperse evenly throughout the chloroplast (Figure 4A, Supplementary Figure S5A). Notably, a subset of *us10c-1*/+ cells (about 7%) displayed chloroplasts with BPG2-GFP forming foci in some, while showing a diffuse distribution in others (Figure 4A–C). Furthermore, nucleoid-unassociated proteins such as BPG2^1-238^-GFP and RbcS1A-GFP exhibited distribution patterns akin to that of BPG2-GFP, either encircling chlorophyll or showing widespread diffusion, in *us10c-1*/+ chloroplasts (Figure 4B, C, Supplementary Figure S5B, C). These findings underline that uS10c is essential for the proper localization of BPG2 within chloroplasts.

As nucleoid organization is dynamic during chloroplast development (12,13), we wondered if the mislocalization of BPG2 results from abnormal nucleoid organization in chloroplasts of *us10c-1*/+. SWI/SNF complex B-2 (SWIB2) is a nucleoid core protein in chloroplasts (15). We analyzed the distribution pattern of SWIB2 and found that, unlike BPG2, SWIB2-GFP still formed foci in most chloroplasts of *us10c-1*/+ (Figure 4D, Supplementary Figure S5D), suggesting that the nucleoid organization might not be affected by the deficiency in uS10c. Consequently, the mislocalization of BPG2 indicates that it fails to target nucleoids when uS10c is deficient.

The failure of BPG2 to target nucleoids could arise from either the inability of BPG2 to associate with ribosomes or the detachment of ribosomes from nucleoids. To determine the underlying cause, the distribution pattern of ribosomes in *us10c-1*/+ chloroplasts was analyzed using the plastid ribosomal protein S5 (referred to as uS5c) as an indicator. The results showed that, as observed in WT, uS5c-GFP formed foci in the chloroplasts of *us10c-1*/+ leaf cells (Figure 4E, Supplementary Figure S5E), indicating that the association between ribosomes and nucleoids was not affected by the lack of uS10c. Therefore, the mislocalization of BPG2 in *us10c-1*/+ chloroplasts results from the disassociation of BPG2 from ribosomes due to the lack of uS10c, consistent with the findings revealed by the sucrose density gradient centrifugation assay.

Next, we examined the impact of the loss of *BPG2* function on the distribution pattern of uS10c. The results showed that in mature leaf cells of *bpg2-2*, the distribution pattern of uS10c in chloroplasts was similar to that observed in WT (Supplementary Figure S5F). However, in seedling cotyledon cells during de-etiolation, the dynamic distribution of uS10c was affected by the loss of BPG2 function. Specifically, in both WT and *bpg2-2* backgrounds, uS10c was stacked on one side of the chloroplast or formed into a ring shape attached to the inner envelope of chloroplasts during the initial two days after germination, mimicking the distribution pattern of nucleoids (Figure 4F, G). By 3 DAG, uS10c formed puncta in most cotyledon cells in WT (Figure 4G, H). In contrast, in *bpg2-2*, only a small portion of cells (less than 30%) displayed punctate uS10c-GFP signals in chloroplasts even at 4 DAG (Figure 4G, H). Until 5 DAG, the distribution pattern of uS10c in *bpg2-2* shows a similar output as observed in WT (Figure 4G, H). We further tested whether the dynamic distribution pattern of uS10c was correlated with the dynamic organization of nucleoids. We again employed SWIB2 as a marker to indicate the nucleoid distribution pattern. As expected, the dynamic distribution pattern of SWIB2-GFP in *bpg2-2* resembled the observations for uS10c (Supplementary Figure S6). These results reveal a tight association between ribosomes and nucleoids.

### uS10c and BPG2 function similarly in rRNA processing

**Figure 5.**
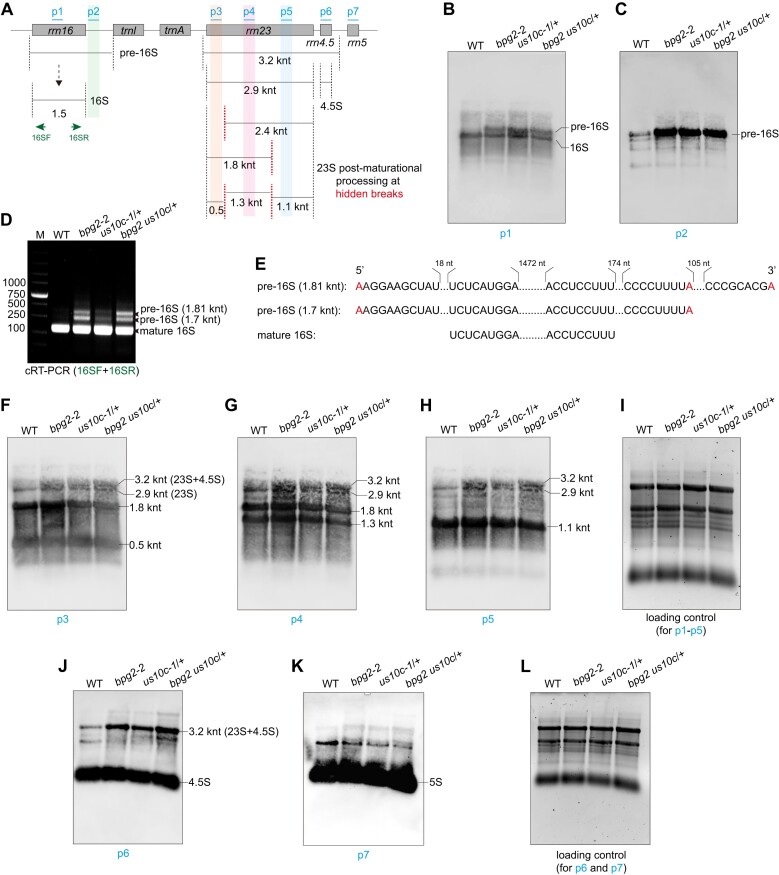
RNA gel blot analysis of the impact of *bpg2-2* and *us10c-1/+* mutations on chloroplast rRNA processing. (**A**) Diagram of the chloroplast *rrn* operon, showing the major accumulating transcripts for each *rrn*. Red dotted lines indicate the positions of hidden breaks in the 23S rRNA. The probes (p1–p7) used for RNA gel blot analysis are marked above the diagram with short blue lines. ‘knt’ stands for kilo nucleotides. (B and C) RNA gel blot analysis of the accumulation levels of 16S rRNAs in WT, *bpg2-2*, *us10c-1*/+ and the double mutant *bpg2 us10c*/+ using probes p1 (**B**) and p2 (**C**). ‘Pre-16S’ refers to 16S rRNA precursors. (**D**) cRT-PCR analysis of the accumulation levels of pre-16S rRNAs in WT, *bpg2-2*, *us10c-1*/+ and *bpg2 us10c*/+. The 1.81-knt and 1.7-knt precursors, along with the mature 16S rRNA, were detected and indicated with arrowheads. (**E**) Sequence information for the 1.81-knt and 1.7-knt intermediates of the 16S rRNA, with affected processing sites highlighted in red font. (F–H) RNA gel blot analysis of the accumulation levels of 23S rRNA intermediates in WT, *bpg2-2*, *us10c-1*/+, and *bpg2 us10c*/+ with probes p3 (**F**), p4 (**G**) and p5 (**H**). Probe p3 detected the 3.2-knt, mature 23S (2.9-knt), 1.8-knt and 0.5-knt transcripts. Probe p4 detected the 3.2-knt, mature 23S (2.9-knt), 1.8-knt, and 1.3-knt transcripts. Probe p5 detected the 3.2-knt, mature 23S (2.9-knt), and 1.1-knt transcripts. The 1.8-knt, 1.3-knt, 1.1-knt, and 0.5-knt bands indicate the products of 23S rRNA following hidden breaks. (**I**) The loading control for RNA gel blot analysis probed with probes p1–p5. 2 μg of RNA was loaded for each sample. (J and K) RNA gel blot analysis of the accumulation level of 4.5S rRNA (**J**) and 5S rRNA (**K**) using probes p6 and p7, respectively. (**L**) The loading control for RNA gel blot analysis probed with probes p6 and p7. 2 μg of RNA was loaded for each sample.

Previous studies have shown that the loss of *BPG2* function affects 16S rRNA maturation in chloroplasts (27,36). Given that uS10c is essential for the interaction of BPG2 with the 30S ribosomal subunit, it is expected that a deficiency in uS10c would produce comparable or broader effects on 16S rRNA processing compared to the loss of BPG2 function. Indeed, the results of RNA gel blot analysis with the p1 probe (specific for 16S rRNA) revealed a reduction in mature 16S rRNA levels and an increase in pre-16S rRNA levels in *bpg2-2*, *us10c-1*/+, and the double mutant when compared to WT (Figure 5A, B). Using the p2 probe, specific for pre-16S rRNAs, we confirmed that the pre-16S rRNA levels were elevated in these mutants (Figure 5A, C). To identify these pre-16S rRNAs, we performed circular reverse transcription-PCR (cRT-PCR), which allowed us to examine both the 5′ and 3′ ends of transcripts. In these mutants, two pre-16S rRNA bands were predominantly detected, with their levels relatively lower in *us10c-1*/+ (Figure 5D). Sequencing revealed that these two bands corresponded to the 1.81-knt (1816 nt) and the 1.7-knt (1702 nt) precursors, respectively (Figure 5E). Notably, both precursors shared an identical 5′ end, suggesting that at least one and two processing steps were influenced at the 5′ and 3′ ends, respectively, during the 16S rRNA maturation in these mutants.

We subsequently analyzed the impact of *bpg2-2* and *us10c-1*/+ mutations on 23S rRNA processing. RNA gel blot results with 23S rRNA-specific probes, including p3, p4, and p5, revealed an increase in the accumulation level of the 3.2-knt bicistronic precursor (consisting of 23S and 4.5S rRNAs) in *bpg2-2*, *us10c-1*/+ and the double mutant (Figure 5F-I). However, no clear difference was observed in the level of mature 23S rRNA (the 2.9-knt band) between the mutants and WT. The 23S rRNA normally undergoes ‘hidden breaks’ through two alternative pathways following maturation: one break occurs near the 5′ end, resulting in the production of the 0.5-knt and 2.4-knt transcripts; another break produces the 1.8-knt and the 1.1-knt transcripts. The 2.4-knt and 1.8-knt transcripts undergo breaks again to produce the final 0.5-knt, 1.3-knt and 1.1-knt transcripts as illustrated in Figure 5A (45). According to the results obtained with the p3 (Figure 5F) and p4 (Figure 5G) probes, no significant differences in the accumulation levels of the 0.5-knt, 1.8-knt and 1.3-knt transcripts were observed among the tested genotypes. A similar outcome was also observed for the 1.1-knt transcript, as detected with the p5 probe (Figure 5H).

Using the p6 probe (specific for 4.5S rRNA), we confirmed that the levels of the 3.2-knt precursor were increased in mutants, while no notable difference was observed in the level of mature 4.5S rRNA between WT and mutants (Figure 5J). Additionally, as detected by the p7 probe (specific for 5S rRNA), the accumulation level of 5S rRNA was not affected by either the loss of BPG2 function or the heterozygous deletion of *uS10c* (Figure 5K, with the loading control shown in Figure 5L).

These findings indicate that uS10c and BPG2 function similarly in the processing of both 16S and 23S-4.5S pre-rRNAs, though their involvement in other rRNA processing steps is limited.

### *bpg2-2* and *us10c-1*/+ produce similar impacts on protein accumulation in chloroplasts

**Figure 6.**
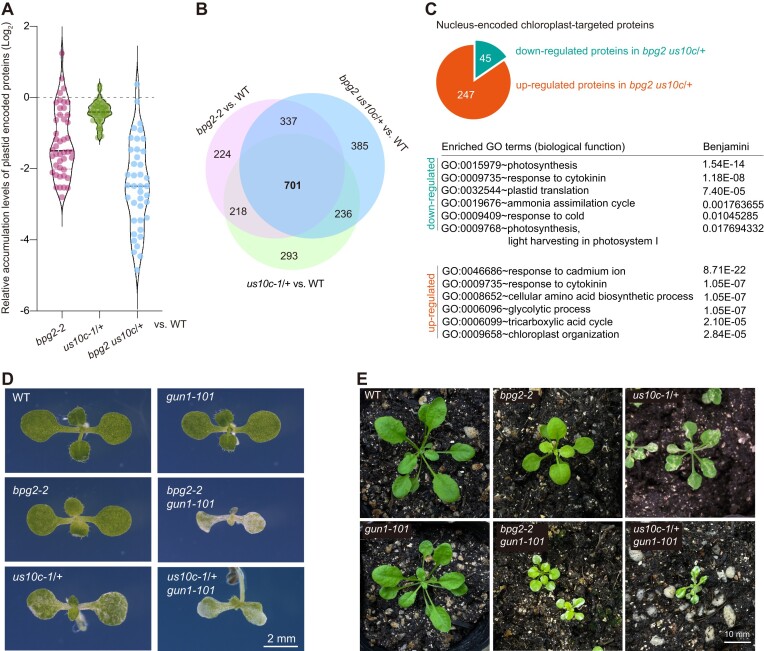
The impacts of *bpg2-2* and *us10c-1/+* mutations on chloroplast protein accumulation and plant growth in the *gun1* mutant background. (**A**) Comparison of relative accumulation levels of plastid-encoded proteins among *bpg2-2*, *us10c-1*/+ and *bpg2 us10c*/+ based on DIA-MS results. The relative accumulation level of each protein was determined by the value of log2[fold change (FC), mutants vs. WT]. For more details, see Supplementary Dataset S2. Significance analysis was performed using the Student's *t*-test. (**B**) A Venn diagram shows the intersection of significantly affected proteins in *bpg2-2*, *us10c-1*/+ and *bpg2 us10c*/+ relative to WT (|FC| > 1 and *P* < 0.05), based on DIA-MS results. For more details, see Supplementary Dataset S3. (**C**) GO enrichment analysis of significantly affected nucleus-encoded plastid-targeted proteins in *bpg2 us10c*/+. Among these proteins, 247 were upregulated, while 45 were downregulated in *bpg2 us10c*/+. GO terms related to biological function are ranked by adjusted *P* values (Benjamini). (**D**) Shoot phenotypes of WT, *bpg2-2*, *us10c-1*/+, *gun1-101* and the double mutants *bpg2-2 gun1-101* and *us10c-1/+ gun1-101* grown on solid 1/2 MS medium at 8 DAG. The scale bar represents 2 mm. (**E**) Phenotypes of the aforementioned plant genotypes grown in soil at 25 DAG. The scale bar represents 10 mm.

We further analyzed the impact of *bpg2-2* and *us10c-1*/+ mutations on chloroplast protein abundance using DIA-MS. In Arabidopsis, the plastome encodes about 100 proteins. Our DIA-MS experiment identified 44 plastome-encoded proteins in 3-day-old seedlings (Supplementary Dataset S2). As expected, the accumulation levels of most of these proteins were reduced in *bpg2-2*, *us10c-1*/+ and the double mutant (Figure 6A), which was closely associated with impaired ribosome biogenesis.

In addition to the plastome-encoded proteins, the plastid proteome comprises over 3000 nucleus-encoded plastid-targeted proteins. Their expression is typically responsive to plastid-to-nucleus signaling, adapting to various plastid states (46). The DIA-MS results revealed significant alterations in the accumulation levels of 701 proteins in *bpg2-2*, *us10c-1*/+ and the double mutant *bpg2 us10c*/+ (Figure 6B). Among these affected proteins, 292 were nucleus-encoded plastid-targeted proteins (Supplementary Dataset S3). Notably, in *bpg2 us10c*/+, the majority of these proteins were upregulated, with only 45 exhibiting downregulation (Figure 6C). Gene Ontology (GO) enrichment analysis revealed that the downregulated proteins were primarily associated with photosynthesis, whereas the upregulated proteins were implicated in signaling responses, metabolic processes, and chloroplast organization (Figure 6C).

GENOME UNCOUPLED 1 (GUN1) is essential for tuning plastid proteome composition by coordinating the nuclear gene expression with diverse plastid states (47–49). Considering the significant changes in the plastid proteome composition observed in both *bpg2-2* and *us10c-1*/+, we investigated the impact of blocking GUN1 function on plant growth in these two mutants. To address this, we introduced *gun1-101* (a knockout allele of *GUN1*) into the *bpg2-2* and *us10c-1*/+ mutant backgrounds by crossing. Under normal growth conditions, most *gun1-101* plants exhibit a normal growth phenotype (Figure 6D, E). However, deleting *GUN1* function in *bpg2-2* and *us10c-1*/+ resulted in severely pale and variegated shoot phenotypes during the seedling stage (Figure 6D). By 25 DAG, the shoots of *bpg2-2 gun1-101* and *us10c-1*/+ *gun1-101* were not only variegated but also smaller compared to their single mutant counterparts (Figure 6E). Furthermore, progenies could not be obtained from either *bpg2-2 gun1-101* or *us10c-1*/+ *gun1-101*, as these plants failed to reach the reproductive growth stage.

Collectively, these observations indicate that the loss of *BPG2* function and *uS10c* haploinsufficiency elicit comparable impacts on chloroplast protein accumulation, and both induce a GUN1-dependent plastid signaling response.

## Discussion

In this study, we investigated how BPG2, a nucleoid-associated YqeH-type GTPase, targets ribosomes to explore the relationship between nucleoids and ribosome biogenesis in chloroplasts. YqeH belongs to the circularly permuted GTPase family with an atypical G-domain motif order of G4-G5-G1-G2-G3 in the C-terminal region (22,50). A previous study demonstrated that all these G-domain motifs are essential for the biological function of BPG2 (27). The results of the present study further revealed that the GTPase domain is required for BPG2 to target ribosomes by interacting with RP uS10c. This interaction, in turn, determines the association of BPG2 with nucleoids. In the N-terminal region, YqeH typically features a Zn-finger domain, which is predicted to be involved in RNA binding (51). Indeed, both human MTG3 (a mitochondrial YqeH homolog) and bacterial YqeH exhibit non-specific RNA-binding activity (26,29). In the case of *E. coli*, the Zn-finger domain is essential for YqeH to interact with the 30S ribosomal subunit (26). In Arabidopsis, although our results here indicate that the Zn-finger domain is not responsible for the association of BPG2 with nucleoids, a previous study reported that mutations in this domain result in the loss of BPG2 function (27). These findings together suggest that the role of YqeH in ribosome biogenesis requires both its non-specific RNA binding, facilitated by the Zn-finger domain, and its interaction with specific ribosomal proteins mediated by the GTPase domain.

In both bacteria and mitochondria, YqeH is found to be exclusively associated with the small ribosomal subunit (26,28,29). Our results also revealed that BPG2 is exclusively present in 30S ribosomal fractions. In yeast mitochondria, YqeH has been detected in protein complexes pulled down by RP uS5m (52). Similarly, in human mitochondria, tandem affinity purification analysis has unveiled the association of MTG3 with RPs from the small subunit (29). However, the precise interacting partner of YqeH in ribosomes remains unidentified in both bacterial and mitochondrial contexts. Our findings suggest that BPG2 and uS10c likely form a transient molecular module involved in rRNA processing during 30S ribosome biogenesis in chloroplasts. It is worth noting that uS10m, the orthologue of RPS10 in human mitochondria, is part of the MTG3 interaction network (29). Nevertheless, due to the structure divergence between mitochondrial and chloroplastic ribosomes (39,53), whether uS10m is involved in the association between MTG3 and ribosomes remains to be further investigated.

In chloroplasts, uS10c is part of the core particle of 30S ribosomal subunits due to its strong binding activity with 16S rRNA (54). A previous study on migration profiling of chloroplast macromolecular complexes revealed that uS10c, along with ten other RPs, is enriched in a lower molecular weight fraction, contrasting with other RPs such as bS1c (55). Our findings here demonstrate that uS10c is embedded into pre-30S ribosomal particles when 16S rRNA is relatively exposed. These observations strongly suggest that uS10c functions as a foundational component during the early assembly stages of the 30S ribosome within chloroplasts. However, in *E. coli*, uS10 is loosely associated with the rRNA-protein complex and acts as a tertiary binding protein, incorporated into ribosomes at later stages, although it is required for cell viability (1,54). In mammalian mitochondria, uS10m also belongs to the late assembly groups during ribosome biogenesis, whereas bS1m is integrated into ribosomes at an earlier stage (56). The divergence suggests that the role of uS10/Rps10 homologs in ribosome assembly is not conserved evolutionarily.

Our results indicate that the uS10c-BPG2 module-mediated ribosome biogenesis steps occur in chloroplast nucleoids. In *E. coli*, nascent pre-16S rRNA colocalizes with nucleoids in an RNase III-dependent manner (8). In mammalian mitochondria, newly synthesized RPs tend to be enriched in the vicinity of nucleoids in a transcription-dependent manner (9). These observations suggest that certain steps of ribosome assembly take place in association with nucleoids in both prokaryotes and prokaryote-derived organelles. However, it remains unclear whether the mechanism by which the pre-ribosomal particles interact with nucleoids is conserved across these systems. In prokaryotic cells, transcription and translation processes can be coupled, known as co-transcriptional translation, serving as a potential mRNA quality control mechanism that prevents premature transcription termination (57,58). Previous studies have revealed that, in *E*. *coli*, the association between the RNA polymerase and the ribosome is mediated through the interaction between NusG (N-utilization substance G, a transcription elongation factor) and RP uS10 (59). In the human pathogen *Mycoplasma pneumoniae*, the association is mediated through NusA (also a transcription elongation factor) interacting with the mRNA entry site of the ribosome (60). In chloroplasts, a set of RPs and the plastid translation EF-Tu (RAB8D) are detected in TACs of nucleoids (14,15), suggesting that coupling likely occurs in plant chloroplasts. Indeed, a recent study provided evidence that the chloroplast NusG connects ribosomes to the plastid-encoded RNA polymerase complex by interacting with both uS5c and uS10c in nucleoids (61). Based on our observations, the absence of uS10c has a limited impact on the distribution pattern of ribosomes. Therefore, it remains interesting to study further the importance of the interaction between NusG and uS5c for ribosome attachment to nucleoids when uS10c is absent.

At the physiological level, the loss of BPG2 function affects chloroplast development but is not fatal, whereas uS10c is indispensable for plant viability. Our results further reveal that *uS10c* is a haploinsufficient gene. Haploinsufficiency, which is common in diploid organisms, occurs when the fitness of the organism is compromised by heterozygous deletion of certain essential genes (43). Moreover, it has been found that most haploinsufficient effects, exhibited under optimal growth conditions, can be alleviated by slowing the growth rate in yeast (62), revealing that haploinsufficiency is highly context-dependent. Consistent with this phenomenon, several representative phenotypes, including leaf variegation, deformity, and chlorophyll aggregation resulting from the heterozygous deletion of *uS10c*, are alleviated by blocking *BPG2* function. We thus hypothesize that reducing ribosome biogenesis in chloroplasts by other means may also mitigate the haploinsufficiency of *uS10c*, which remains an open question for further study. Another interesting observation is that the uS10c protein level is significantly lower in the *bpg2 us10c*/+ double mutant compared to that in the *us10c-1*/+ single mutant. Given that the uS10c protein level is also reduced in the *bpg2-2* single mutant, we believe that both *bpg2*-induced plastid signaling and the heterozygous deletion of *uS10c* contribute to the reduced accumulation of uS10c protein in the *bpg2 us10c*/+ double mutant. This outcome leads to an additive effect on shoot development in *bpg2 us10c*/+, especially at the seedling stage.

Our findings also reveal that mutations in *BPG2* or *uS10c* severely affect the accumulation of most plastome-encoded proteins, which may greatly affect the state of chloroplasts. Since the nuclear genome encodes most plastid proteins, plants have evolved a plastid/chloroplast-to-nucleus communication mechanism to adapt to various plastid/chloroplast states. GUN1 is an important factor that mediates multiple plastid signaling pathways (47–49). Our findings reveal that plants cannot tolerate the combined loss of *GUN1* function with either the loss of *BPG2* function or the heterozygous deletion of *uS10c*, especially during the transition from vegetative to reproductive growth stages. Consistent with our observations, previous studies have revealed that GUN1 becomes essential when plastid translation is compromised by factors such as the knockdown of plastid EF-Tu (34), loss of function in *Plastid Ribosomal Protein L11* (63), and treatment with lincomycin (a specific inhibitor of plastid translation) (64). These insights underscore the critical role of the uS10c-BPG2 module in constructing the chloroplast proteome.

Moreover, based on the characteristics of other potential interacting partners of BPG2 identified through the Y2H assay in this study, BPG2 is probably involved in multiple biological processes beyond its role in uS10c-mediated ribosome biogenesis in chloroplasts. For instance, among the potential partners of BPG2, Phosphatidylglycerophosphate Phosphatase 1 (PGPP1) plays a role in the synthesis of phosphatidylglycerol, a fundamental component of thylakoid membranes. The loss of PGPP1 function results in a pale-green shoot phenotype, similar to mutants lacking BPG2 (65,66). However, unlike BPG2, PGPP1 does not localize to nucleoids but is diffused throughout the chloroplasts (65), revealing that the role of BPG2 in nucleoids is not associated with PGPP1. PSA2 is also identified as a potential interacting partner of BPG2. PSA2 is a thylakoid lumen-localized protein-disulfide reductase (67) with a foci-like distribution pattern in chloroplasts (68). The loss of PSA2 function affects the accumulation of proteins associated with photosystem I (PSI) but not those associated with PSII (67). Our DIA-based proteomics analysis indicates that the loss of BPG2 function impacts both PSI and PSII. This raises an intriguing possibility that BPG2 directly influences PSI protein accumulation through its interaction with PSA2.

**Figure 7.**
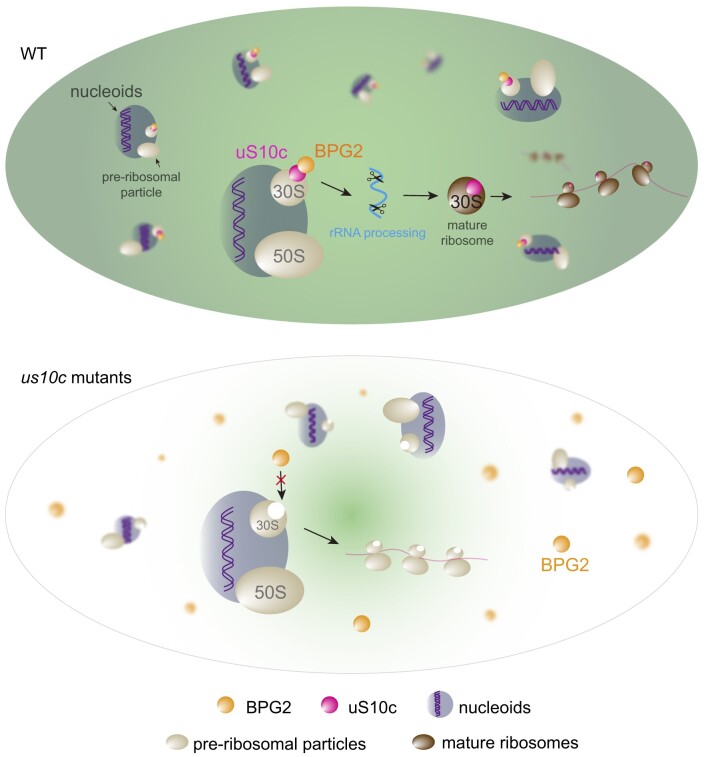
A model for the uS10c-mediated association of BPG2 with ribosomes in chloroplast nucleoids. In WT cells, the YqeH-type GTPase BPG2 functions as a chloroplast RBF that targets pre-30S ribosomal particles for rRNA processing in chloroplast nucleoids. In this process, ribosomal protein uS10c mediates the interaction between BPG2 and the ribosomes. In the absence of uS10c (as observed in the *us10c* mutant), BPG2 is unable to target nucleoid-associated pre-ribosomal particles, resulting in its diffusion throughout the chloroplast stroma. As a result, ribosome biogenesis and maturation are disrupted, thereby impacting plastid protein production.

Based on our observations, we devised a model to illustrate the uS10c-mediated association of BPG2 with ribosomes (as shown in Figure 7). In the chloroplasts of WT cells, BPG2 binds to pre-30S ribosomal particles within nucleoids through its interaction with RP uS10c via the C-terminal GTPase domain, which ensures efficient processing of pre-rRNA and the production of plastome-encoded proteins. Conversely, a deficiency or dysfunction in uS10c disrupts the BPG2-ribosome interaction, leading to impaired targeting of BPG2 to nucleoids and reduced rRNA processing efficiency.

## Supplementary Material

gkae339_Supplemental_Files

## Data Availability

Both DDA-MS and DIA-MS raw data are available via ProteomeXchange under the identifier PXD027861.
