# Supplementary material for: The uS10c-BPG2 module mediates ribosomal RNA processing in chloroplast nucleoids
Source: Nucleic Acids Res. 2024 Apr 30;52(13):7893–909. doi: 10.1093/nar/gkae339 (PMC11260468; doi:10.1093/nar/gkae339)
Supplement: gkae339_Supplemental_Files [file gkae339_supplemental_files.zip › Supplementary Figures and tables.pdf]

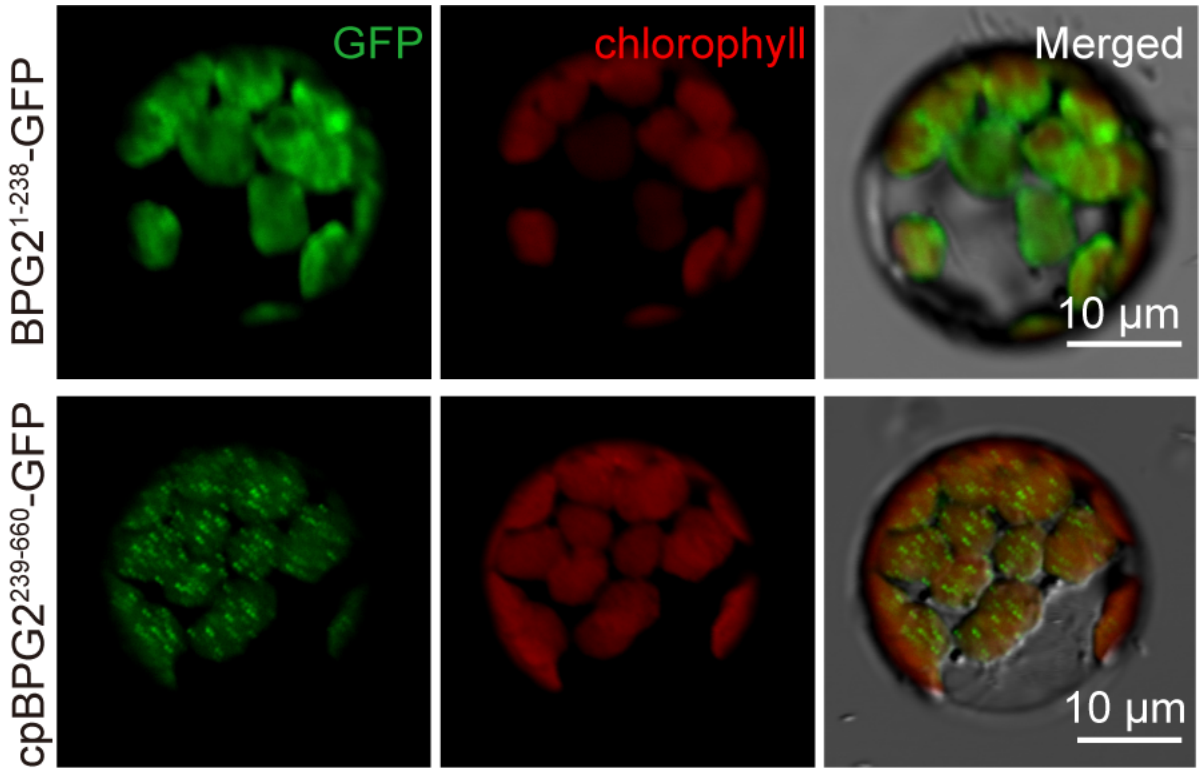


**Figure S1. Distribution pattern analysis of the truncated BPG2 proteins in chloroplasts.** The upper panel shows the distribution pattern of $BPG2^{1\text{-}238}$ which contains the Zn-finger domain. The lower panel shows the distribution pattern of $cpBPG2^{239\text{-}660}$ which contains the GTPase domain and is fused with the chloroplast transit peptide of RbsS1A at the N terminus.

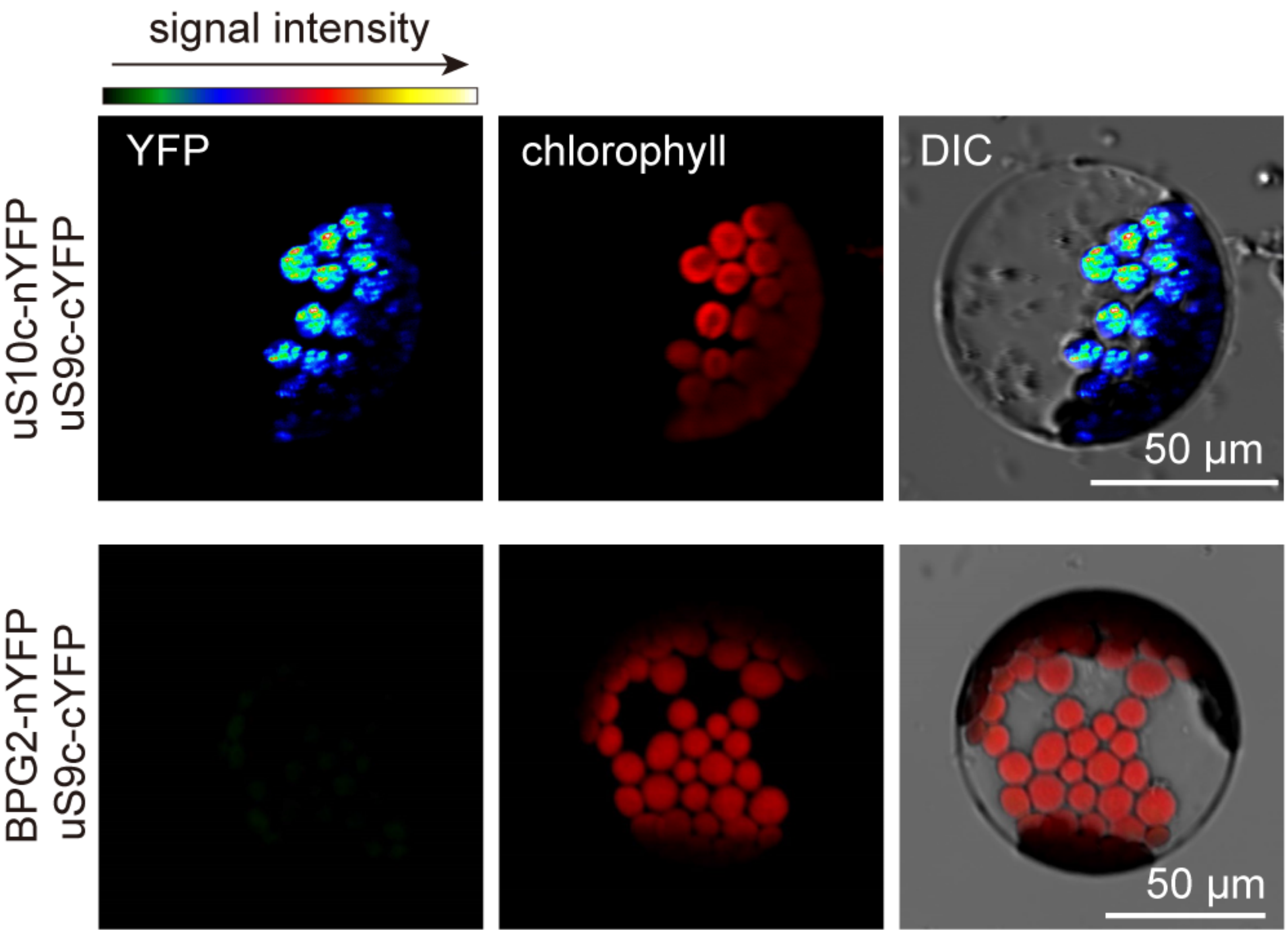


**Figure S2. BiFC analysis of uS10c-uS9c and BPG2-uS9c interactions.**

The upper panel shows the BiFC analysis for the interaction between uS10c and uS9c. The lower panel shows the BiFC analysis for the interaction between BPG2 and uS9c. A heatmap indicates the intensity of YFP fluorescence signal.

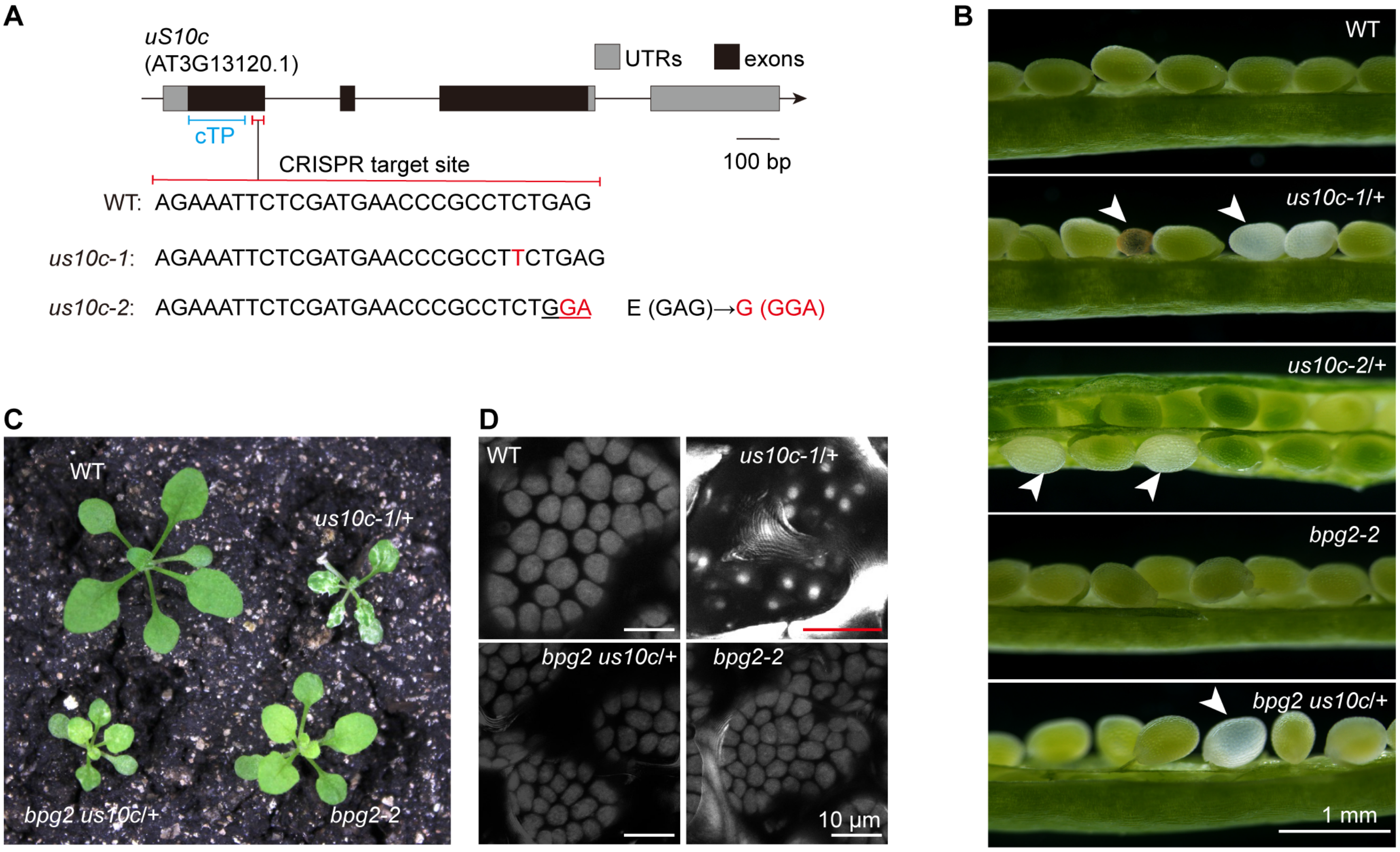


**Figure S3. Impacts of *uS10c* mutations on seed development and chlorophyll distribution in rosette leaves.**

**A**, Mutation sites in *us10c-1* and *us10c-2*. The target site for Cas9 is in the first exon of the *uS10c* gene, which is adjacent to the 3' end of the sequence encoding the chloroplast transit peptide (cTP).

**B**, Seed phenotypes within green siliques of WT, *us10c-1*/+, *us10c-2*/+, *bpg2-2*, and *bpg2 us10c*/+ at 12 days post-pollination. White arrowheads indicate aborted or albino seeds.

**C**, Shoot phenotypes of WT, *us10c-1*/+, *bpg2-2*, and *bpg2 us10c*/+ at 20 DAG.

**D**, Chlorophyll distribution patterns in chloroplasts of rosette leaves from WT, *us10c-1*/+, *bpg2-2*, and *bpg2 us10c*/+ at 20 DAG.

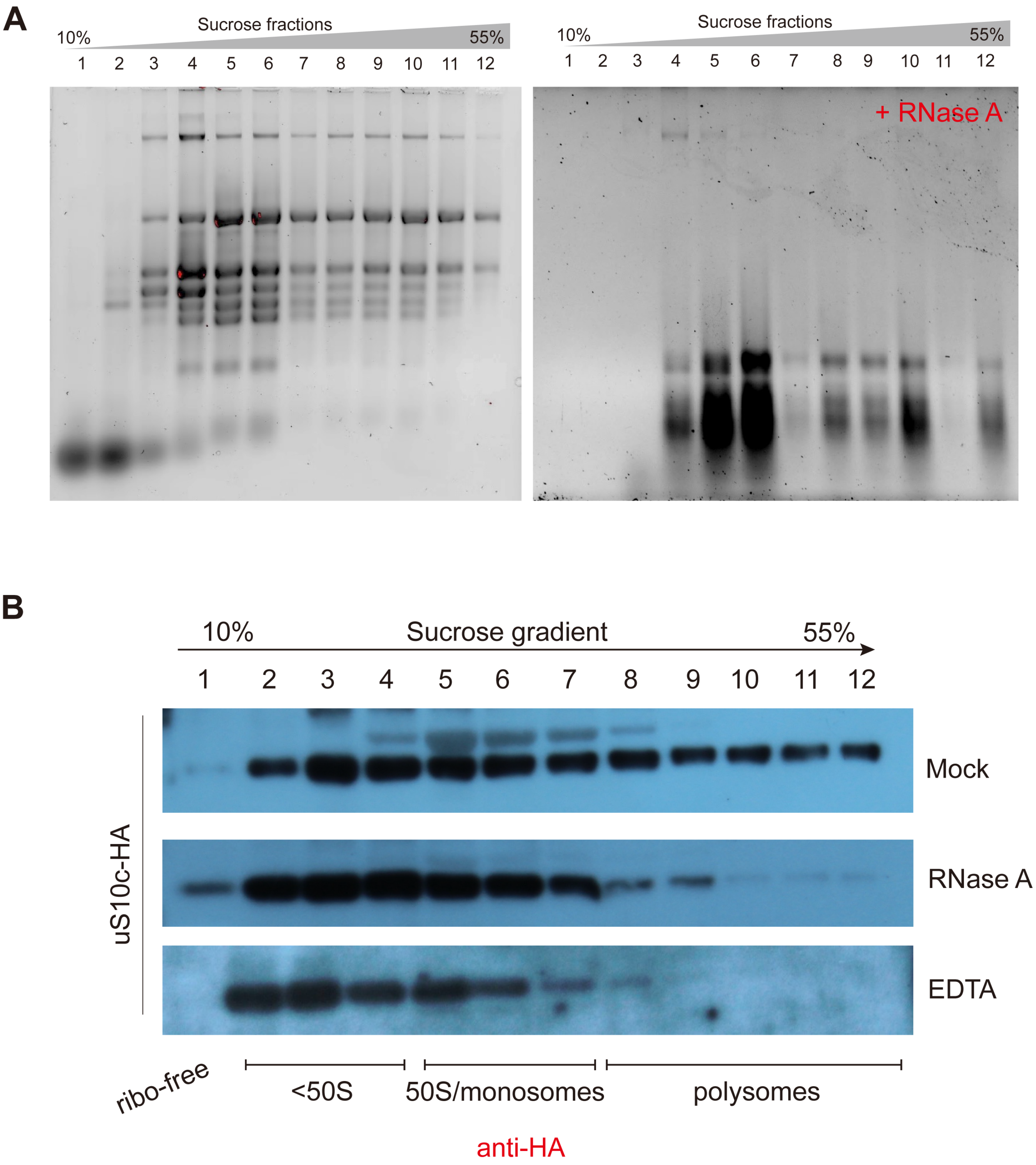


**Figure S4. Analysis of the association between uS10c-HA fusion protein and chloroplast ribosomes.**

**A**, Migration pattern analysis of rRNAs after treatment with or without RNase in sucrose density gradient fractions (10-55%) using gel electrophoresis.

**B**, The migration patterns of uS10c-HA in sucrose density gradient fractions (10-55%). Proteins were extracted from the 35S:uS10c-HA transgenic plants and then treated with or without RNase A and EDTA before centrifugation. For detecting the fusion protein, the specific antibody for the HA tag was employed.

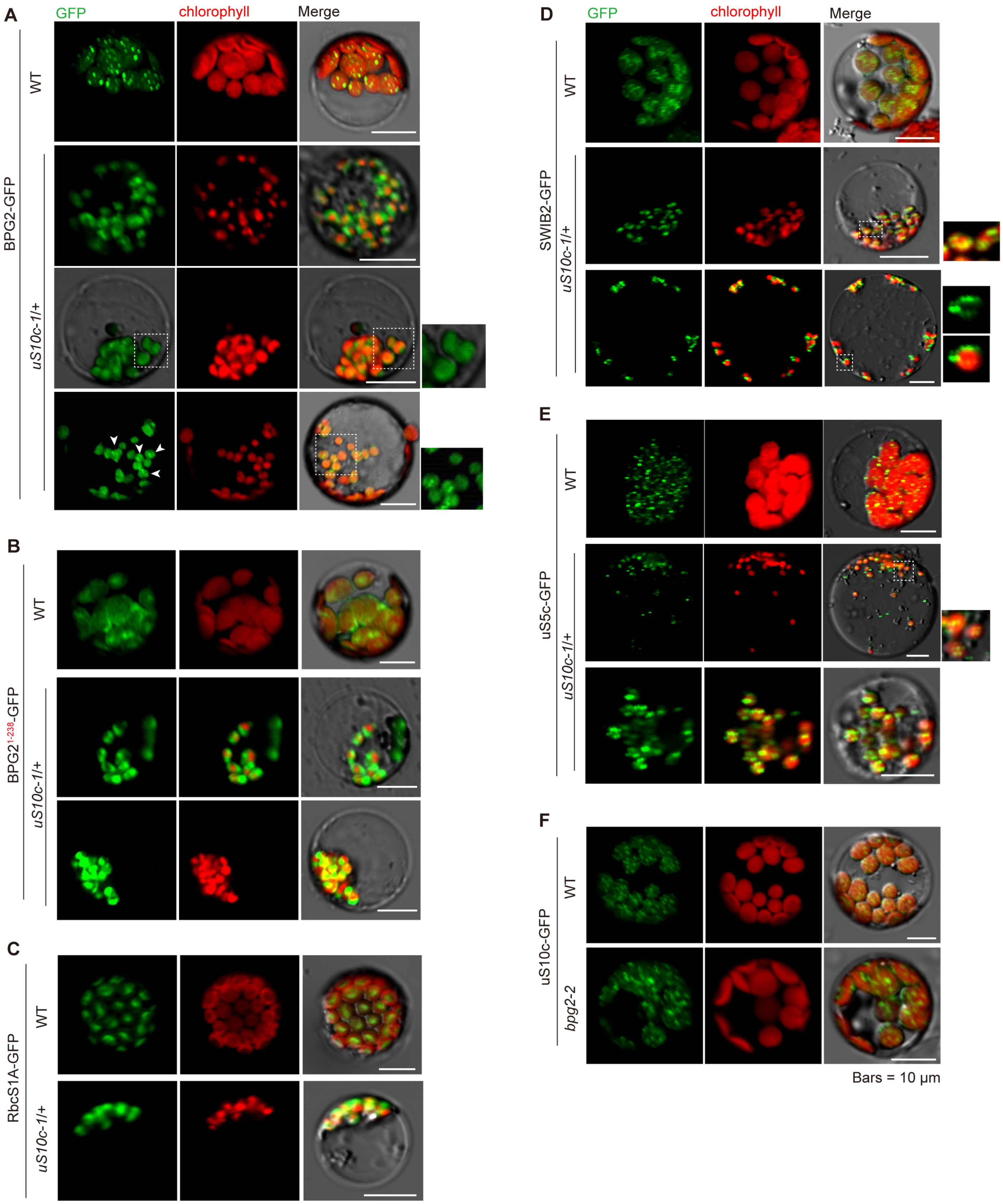


**Figure S5. Protein distribution patterns of BPG2, BPG2$^{1-238}$, RbcS1A, SWIB2, uS5c and uS10c in chloroplasts.**

**A**-**E**, Analysis of distribution patterns of BPG2-GFP (**A**), BPG2$^{1-238}$-GFP (**B**), RbcS1A-GFP (**C**), SWIB2-GFP (**D**), and uS5c-GFP (**E**) in chloroplasts from the WT and *us10c-1*/+ backgrounds.

**F**, Analysis of the uS10c-GFP distribution patterns in the WT and *bpg2-2* backgrounds.

Scale bars in all images represent 10 μm.

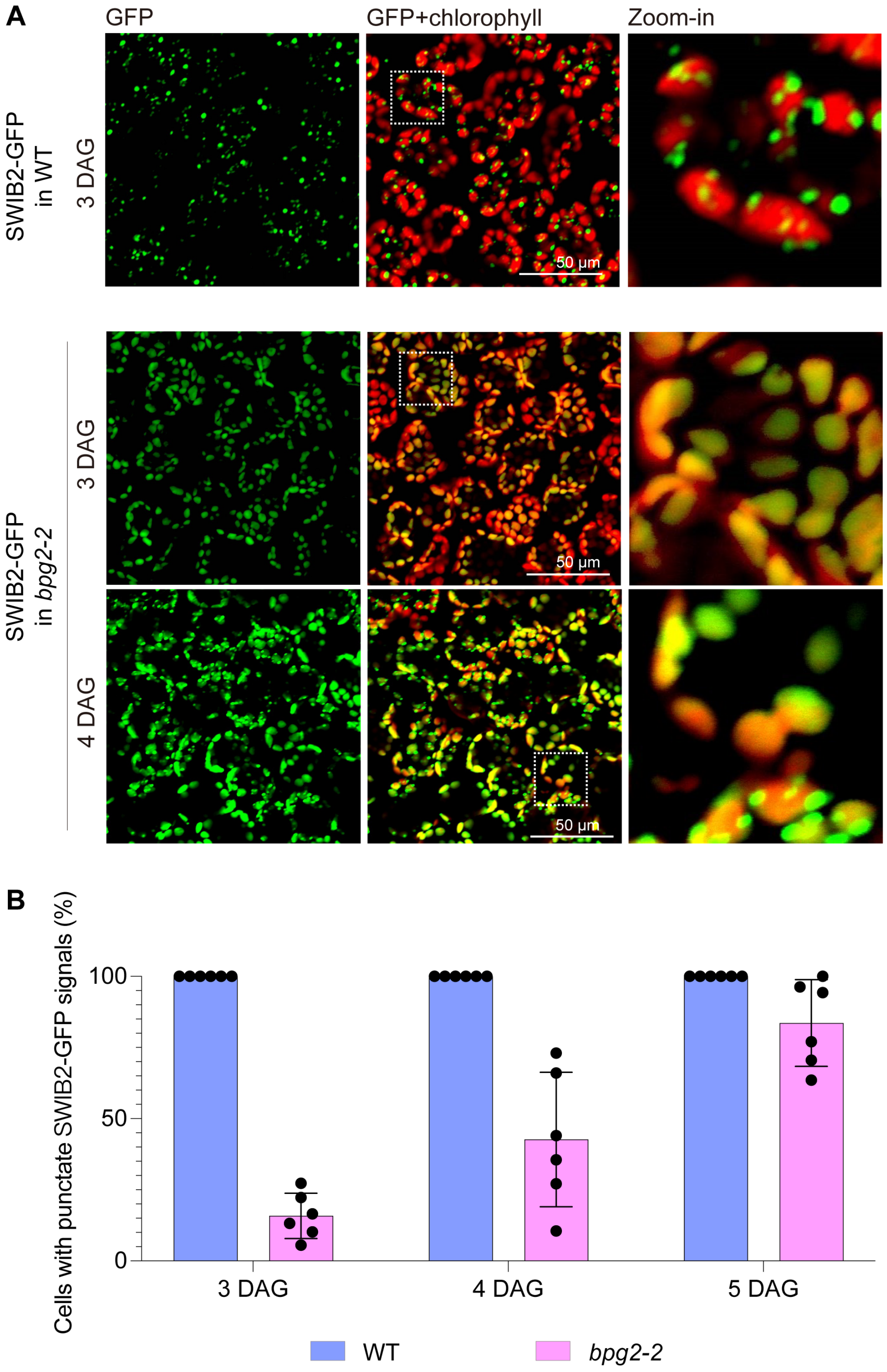


**Figure S6. Loss of BPG2 function affects SWIB2 dynamic distribution in chloroplasts.**

**A**, Distribution patterns of SWIB2-GFP in chloroplasts of WT and *bpg2-2* cotyledons at indicated stages. Zoom-in images were shown for better observation.

**B**, Percentage of cells with punctate SWIB2-GFP organization in chloroplasts of WT and *bpg2-2* at 3~5 DAG.

**Table S1. Potential BPG2 interacting partners screened by the Y2H assay.**

| AGI number | Clones | Description | Subcellular localization |
|---|---|---|---|
| AT3G13120 | 7 | Ribosomal protein S10p/S20e family protein | chloroplast |
| AT3G58830 | 2 | Encodes a phosphatidylglycerophosphate phosphatase that localizes to chloroplasts | chloroplast in shoots mitochondrion in roots |
| AT4G00895 | 1 | ATPase | chloroplast |
| AT2G14260 | 1 | Encodes proline iminopeptidase | chloroplast |
| AT1G43890 | 1 | ARABIDOPSIS RAB GTPASE HOMOLOG B18 | nucleus, plasma membrane |
| AT5G53850 | 1 | Encodes a trifunctional dehydratase/ enolase/phosphatase involved in the methionine salvage | cytoplasm |
| AT1G20070 | 1 | hypothetical protein | mitochondrion |
| AT5G15140 | 1 | PATERNALLY EXPRESSED GENE 9 | cytoplasm |
| AT1G73655 | 1 | FKBP-like peptidyl-prolyl *cis-trans* isomerase family protein | chloroplast |
| AT3G55360 | 1 | Enoyl-CoA reductase | endoplasmic reticulum |
| AT2G34860 | 1 | DnaJ-like zinc finger domain-containing protein which regulates the assembly of photosystem I and seed development | chloroplast |

List of genes encoding the potential interacting partners of BPG2. The number of independent positive clones containing the corresponding gene was shown in the 'clones' column. The description for each gene was according to the information in The Arabidopsis Information Resource (TAIR). The subcellular localization information was according to the Subcellular Location of Proteins in Arabidopsis Database (SUBA) and previously published findings.

## Table S2. Primers used in this study.

| Name | Sequences | Purpose |
|---|---|---|
| BPG2-F | ACGCGTCGACATGGTGGTTTTGATTTCAAGTACA | pBSK-GFP |
| BPG2-N(1-238)-R | CGCGGATCCATCATCATAATTGTCTTTCTTTGC | pBSK-GFP |
| BPG2-R | CGCGGATCCAGCAACACTATCAGAGAGAAAATC | pBSK-GFP |
| BPG2-AD/BD-F | GGGAATTCCATATGATGGTGGTTTTGATTTCAAGTACA | Y2H |
| BPG2-AD/BD-R | CGCGGATCCAGCAACACTATCAGAGAGAAAATC | Y2H |
| uS10c-AD/BD-F | CCGGAATTCATGGCGGTTTCTACTGTATCGTCG | Y2H |
| uS10c-AD/BD-R | cgcGGATCCGAGCTTCACTTCCACATCGACACC | Y2H |
| BPG2$^{1-238}$-BD-R | CGCGGATCCATCATCATAATTGTCTTTCTTTGC | Y2H |
| BPG2$^{239-660}$-BD-F | GGGAATTCCATATGATGGTGACTGTGTGTGCTCGTTGC | Y2H |
| BPG2-YFP-F | cgcGGATCCATGGTGGTTTTGATTTCAAGTACA | BiFC |
| BPG2-YFP-R | acgcGTCGACAGCAACACTATCAGAGAGAAAATC | BiFC |
| uS9c-YFP-F | cgcGGATCCATGGCGTCGATTACGAACCTCGCC | BiFC |
| uS9c-YFP-R | acgcGTCGACACGCTTGGAGAATTGTGGGGC | BiFC |
| uS10c-target | AGAAATTCTCGATGAACCCGCCTCTGAG | CRISPR-Cas9 target site |
| uS10c-MF | ATGGCGGTTTCTACTGTATCGTCGTTT | mutant sequencing |
| uS10c-MR | TATCCGCGTCTACACTGATGGATGAAGAGCT | mutant sequencing |
| uS10c-F | cgcGGATCCATGGCGGTTTCTACTGTATCGTCG | pCAMBIA2300-GFP, BiFC |
| uS10c-R | acgcGTCGACGAGCTTCACTTCCACATCGACACC | pCAMBIA2300-GFP, BiFC |
| pro-uS10cF | cgACGCGTGTTGTCCTCGGTTTCTTGATGTTT | uS10c promoter |
| pro-uS10cR | cggGGTACCTGAATTTTCCCAAAACCACGAATCGAT | uS10c promoter |
| uS10c-HA-R | acgcGTCGACTCAAGCGTAATCTGGAACATCGTATGGGTAGAGCTTCACTTCCACATCGACACC | uS10c-HA vector |
| uS10c-RNAi-F | CCGGAATTCATGGCGGTTTCTACTGTATCGTCG | pHANNIBAL RNAi vector |
| uS10c-RNAi-R | CGGGGTACCCTGGTGTGTCCGGATTTCAAAATG | pHANNIBAL RNAi vector |
| auS10c-RNAi-F | CCCATCGATCTGGTGTGTCCGGATTTCAAAATG | pHANNIBAL RNAi vector |
| auS10c-RNAi-F | CGCGGATCCATGGCGGTTTCTACTGTATCGTCG | pHANNIBAL RNAi vector |
| uS10c-F2 | acgcGTCGACATGGCGGTTTCTACTGTATCGTCG | pBSK-GFP |
| uS10c-R2 | cgcGGATCCGAGCTTCACTTCCACATCGACACC | pBSK-GFP |
| SWIB2-F | ACGCGTCGACATGGCGGTTTCTTCTGGAACATTC | pBSK-GFP/mCherry |
| SWIB2-R | CGCGGATCCGAGGAAGTGAGGACCGATGAGCTT | pBSK-GFP/mCherry |
| RbcS1A-F | acgcGTCGACATGGCTTCCTCTATGCTCTCTTCC | pBSK-GFP |

| | | |
|---|---|---|
| RbcS1A-R | cgcGGATCCACCGGTGAAGCTTGGTGGCTTGTA | pBSK-GFP |
| uS5c-F | acgcGTCGACATGGCGACAGCATCAGCTCTCTCA | pBSK-GFP |
| uS5c-R | cgcGGATCCCTTCCAGAGTTCTTCCATGGGGAT | pBSK-GFP |
| 16S-RTP | GTATTAGCAGCCGTTTCCAG | reverse transcription for cRT-PCR |
| 16S-F | TAATCGCCGGTCAGCCATAC | cRT-PCR |
| 16S-R | TCCCAAGGGCAGGTTCTTAC | cRT-PCR |
| Probe p1 | TTCCGATCTCTACGCATTTCACCGCTCCACCGGAAATTCC | RNA gel blot |
| Probe p2 | GATCCTATCAACTTGTTCCGACCTAGGATAATAAGCTCAT | RNA gel blot |
| Probe p3 | AGGGTGCGGCACTCCACCGCTTCGCCTAGCAGCACGACGC | RNA gel blot |
| Probe p4 | CAGGGACACCTTGCGTCCTTGAACCGATAACCATCTTTCG | RNA gel blot |
| Probe p5 | AGGTCCTGACACAAGGTTAGAATTCTAGCTCTTCCAGAGT | RNA gel blot |
| Probe p6 | ACTTGACACCTATCGTAATGATAAACGGCTCGTCTCGCCG | RNA gel blot |
| Probe p7 | CCGCAGGACCTCCCCTACAGTATCGTCACCGCAGTAGAGT | RNA gel blot |
